# Major shifts at the range edge of marine forests: the combined effects of climate changes and limited dispersal

**DOI:** 10.1038/srep44348

**Published:** 2017-03-09

**Authors:** J. Assis, E. Berecibar, B. Claro, F. Alberto, D. Reed, P. Raimondi, E. A. Serrão

**Affiliations:** 1 CCMAR, Centro de Ciências do Mar, Universidade do Algarve, Campus de Gambelas, 8005-139 Faro, Portugal; 2 Estrutura de Missão para a Extensão da Plataforma Continental (EMEPC), Rua Costa Pinto 165, 2770-042, Paço de Arcos, Portugal; 3 Department of Biological Sciences, University of Wisconsin-Milwaukee, Milwaukee, Wisconsin 53201, USA; 4 Marine Science Institute, University of California, Santa Barbara, California 93106, USA; 5 Department of Biology, University of California, Santa Cruz, California 95064, USA

## Abstract

Global climate change is likely to constrain low latitude range edges across many taxa and habitats. Such is the case for NE Atlantic marine macroalgal forests, important ecosystems whose main structuring species is the annual kelp *Saccorhiza polyschides*. We coupled ecological niche modelling with simulations of potential dispersal and delayed development stages to infer the major forces shaping range edges and to predict their dynamics. Models indicated that the southern limit is set by high winter temperatures above the physiological tolerance of overwintering microscopic stages and reduced upwelling during recruitment. The best range predictions were achieved assuming low spatial dispersal (5 km) and delayed stages up to two years (temporal dispersal). Reconstructing distributions through time indicated losses of ~30% from 1986 to 2014, restricting *S. polyschides* to upwelling regions at the southern edge. Future predictions further restrict populations to a unique refugium in northwestern Iberia. Losses were dependent on the emissions scenario, with the most drastic one shifting ~38% of the current distribution by 2100. Such distributional changes might not be rescued by dispersal in space or time (as shown for the recent past) and are expected to drive major biodiversity loss and changes in ecosystem functioning.

Recent climate change has produced several shifts in the distributions and abundances of species^1^. Climate projections suggest further potential for range shifts and may become one of the most important drivers of biodiversity loss^2^. This is particularly obvious at the low latitude range edges, where small variations beyond the peripheral niche may lead to extinction of local populations^3^.

Maintenance of ecosystem function will likely depend on the persistence of structuring species such as kelps^4^, here defined as large brown algae that can form marine forests and have alternating macroscopic and microscopic generations. These species create complex habitats that provide resources, shelter and nursery grounds for many marine organisms^5^. While kelp forests are naturally resilient systems, the ongoing warming of the oceans is shifting their distribution in many regions^5^,^6^, a process that may severely reduce essential habitats and unbalance important trophic interactions for numerous species.

Understanding the processes driving distributional changes of marine habitat forming species and predicting their biological responses to future climate variability is therefore not only a central subject in ecology, but also in conservation management. Many studies have used ecological niche modelling (ENM) to address such emerging issues^7^,^8^,^9^. These models are deeply rooted in the climatic niche theory, benefiting from a straightforward translation into a wide range of statistical methods, linking species occurrence data with environmental gradients to infer spatiotemporal patterns of distributions^10^.

Environmental factors like nutrients and light availability, seawater temperatures and wave action are well known drivers shaping the distribution and abundance of marine species at multiple spatial and temporal scales^11^. However, because kelps display a complex heteromorphic life cycle that includes a diploid macroscopic sporophyte and a haploid microscopic gametophyte, understanding the responses of this particular ecological group to climate variability can be a challenging task because the environment may act differently on each life stage, and the effects on one stage can affect the abundance of the subsequent one^12^,^13^. The ability to detect such specific responses within the global context is highly dependent on the use of fine-scale environmental data (resolution below tens of kilometres), mostly because the rates of change are far more complex than the simple rise or fall of averaged environmental predictors, with many locations displaying unique trends both spatially and seasonally^14^.

The vulnerability of species to climate change is further determined by their ability to migrate to extant suitable habitats^15^. For sessile marine species, migration is primarily mediated through propagule dispersal, which varies dramatically among species. However, most reconstructions of species ranges dichotomize dispersal as either unlimited or non-existent. This may lead to flawed estimations of the potential use of available habitats because species either use all suitable climatic space (unlimited dispersal scenario) or are unable to track change (no dispersal) and experience local extinctions whenever conditions fall outside tolerance limits^10^. In the same way, the ability of species to persist locally (e.g., as cryptic stages) during periods of unfavourable conditions is not usually considered, resulting in the underestimation of local persistence^15^. Acknowledging these processes is important for reconstructing kelp distributions because they have limited dispersal^16^,^17^ and some of their microscopic life stages may be able to persist during environmental conditions that are unfavourable to the macroscopic phase^18^.

A good model system to understand the processes driving range shifts and to predict the consequences of future climate changes is the Atlantic coast along the Iberian Peninsula and northern Africa. This region, hereafter designated Iberia-Morocco, is an important temperate biogeographic transition zone where a considerable number of marine species reach their cold and warm distributional limits^19^. Furthermore, this region is among the most affected by climate change^20^ and empirical evidence has confirmed that many ecosystem-structuring species have contracted ranges there^21^,^22^,^23^,^24^, with potential cascading consequences for the associated communities. One such species is *Saccorhiza polyschides* (Lightfoot) Batters, an important kelp in NE Atlantic waters, and a main canopy-building species in Iberia-Morocco^6^,^21^,^25^. It has undergone local extinctions and bottlenecks^6^ but the causes and magnitude of extinctions in this broad region have not been addressed.

Here we use regression and simulation techniques to (1) investigate the biologically meaningful factors shaping the range edge of *S. polyschides*, (2) examine the relationship between interannual climate variability and range loss, and (3) estimate persistence and population turnover. These challenging questions were addressed by modelling the ecological niche of *S. polyschides* with fine-scale environmental data, and by reconstructing range dynamics for the period 1986–2100 using simulations of potential dispersal and arrested development of microscopic life stages.

## Results

Along the Iberia-Morocco region, the presence and absence of *S. polyschides* was recorded during 197 coastal surveys (retrieving 129 unique gridded cells for the 5 years of field surveys). This dataset was enhanced with 312 historical records compiled from the available literature (198 unique gridded cells since 1986; Supplementary Figure S1, Supplementary Table S1).

### Factors shaping the range edge of *Saccorhiza polyschides*

**Figure 1:**
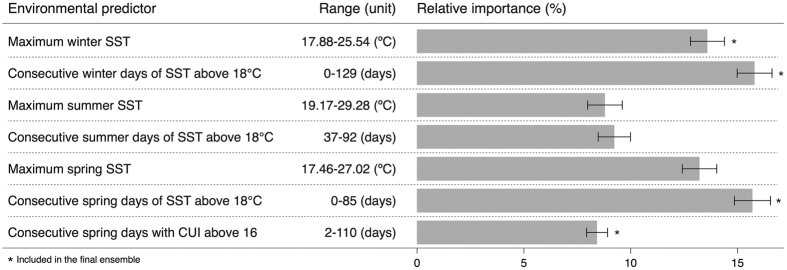
Relative importance of each environmental predictor used for modelling the distribution of ***Saccorhiza polyschides*** **(only contributions above 5% are shown; for more information please refer to Supplementary Table S2)** . Name of predictors (SST - Sea Surface Temperature; CUI - Coastal Upwelling Index; Winter: NDJF; Spring: MAMJ; Summer: JAS), range in the study region, units and mean relative contribution to the accuracy of models (asterisks show predictors included in the final ensemble) are shown.

The transferrable models using data from 2010 and 2012 explained most of the spatial variation of *S. polyschides* (mean deviance: 0.19 ± 0.02) and allowed predicting suitable habitats with minimum areas (0.48 ± 0.02 mean proportion of total area predicted) while retaining high sensitivity (true presence rate) scores >0.9. The most important predictors included in the final ensemble were the number of consecutive winter and spring days with temperatures above 18 °C (15.82% ± 0.82 and 15.72% ± 0.85 contribution, respectively; Fig. 1; Supplementary Table S2), and the maximum winter temperatures (13.61% ± 0.82). The consecutive spring days with favourable upwelling conditions (8.44% ± 0.47; Fig. 1), the maximum winter waves and the consecutive spring days with waves above 3 m were also included in the final ensemble, although the latter two with small contribution to the models (0.39% ± 0.35 and 0.25% ± 0.38, respectively; Supplementary Table S2).

**Figure 2:**
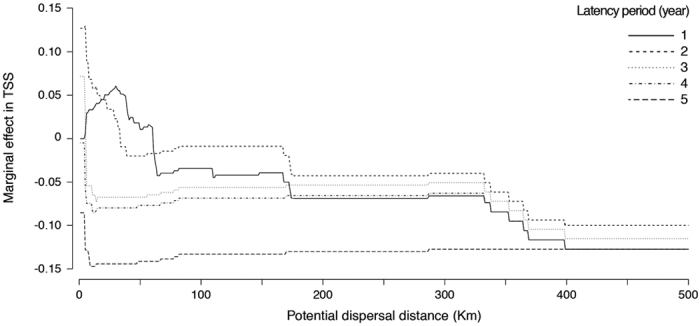
Simulation of the effect of potential dispersal distance (step 1 km) and latency period (step 1 year) on the marginal value of True Skill Statistics (TSS), starting from a state of no dispersal and no latency capacity.

Simulations showed that a maximum dispersal distance of 5 km per year and a maximum latency period of 2 years (gametophytes) produced the highest marginal value in True Skill Statistics (TSS; Fig. 2), an index considering both sensitivity and specificity (true absence rate), and ranging between −1 to +1 (TSS > 0.8 indicate an excellent agreement between modelled and observed records^26^). Using these specific parameters, the TSS of our predictions increased 0.13 units from a reference point of 0.79 for no dispersal and no latency period. The reconstructions made with increasing dispersal distances and latency periods decreased TSS to 0.65 and 0.63, respectively (Fig. 2); thus, further analysis and discussion will focus mainly on the most accurate reconstruction of *S. polyschides* distribution.

### The range edge of *S. polyschides*

**Figure 3:**
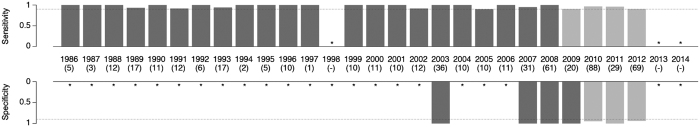
Accuracy of hindcasting (1986–2014) given by sensitivity (true presence rate) and specificity (true absence rate). Dark grey bars show the independent historical records outside the training window of the models (i.e., ground-truthing data). Amount of occurrence data (presences or absences) shown in parenthesis (asterisks indicate the years with no data). Horizontal dotted line depicting the 0.9 threshold.

**Figure 4:**
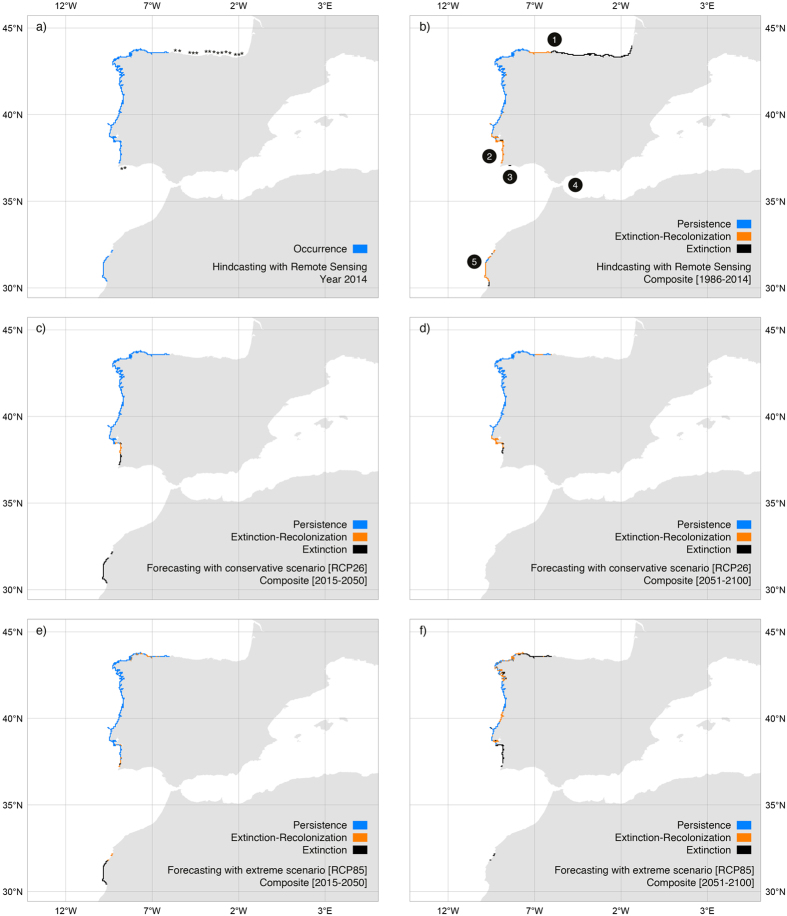
Distribution of *Saccorhiza polyschides* in Iberia-Morocco predicted with remote sensing data (period 1986–2014) and AOGCMs data (period 2015–2100, under two scenarios of greenhouse gas emissions. RCP26 and RCP8.5). (Panel a) Predicted distribution in 2014 and sites (asterisks) where the species occurred in the past but our most recent surveys (years 2010 and 2012) failed to record it. Areas of persistence, extinction and extinction followed by recolonization for the periods (panel b) 1986–2014, (panels c,e) 2015–2050 and (panels d,f) 2051–2100. The reconstruction of distributions integrated a dispersal distance of 5 km and latency period of 2 years. Numbers represent Regions Of Interest: ROI 1: Cabo de Peñas; ROI 2: Carrapateira; ROI 3: Lagos; ROI 4: Tarifa; ROI 5: western Morocco. Maps generated with QGIS (QGIS Development Team, 2016. QGIS Geographic Information System. Open Source Geospatial Foundation Project. http://www.qgis.org/).

**Figure 5:**
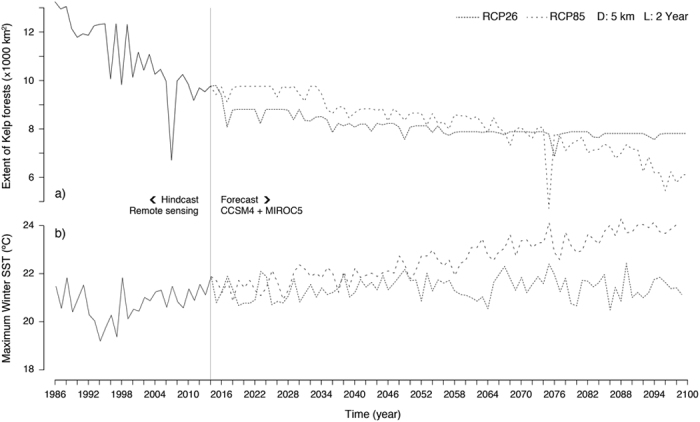
(Panel a) Extent of *Saccorhiza polyschides* in Iberia-Morocco predicted with remote sensing data (period 1986–2014) and AOGCMs data (period 2015–2100, under two scenarios of greenhouse gas emissions; RCP26 and RCP8.5). The models integrated a dispersal distance (D) of 5 km and a latency period (L) of 2 years. (Panel b) Variation of the maximum winter sea surface temperature (SST) averaged for Iberia-Morocco (for additional environmental predictors please refer to Supplementary Figure S5).

By integrating the best dispersal and latency parameters (D = 5 km; L = 2 year) the reconstruction of distributions produced an excellent accuracy^26^, both when validated with all distributional records (n = 509; TSS: 0.92; sensitivity: 0.96; specificity: 0.97) and with the independent dataset prior to 2010 (Fig. 3; n = 323; sensitivities >0.90; specificities: >0.92). This allowed verifying that from 1986 to 2014*S. polyschides* had its widest distribution from the Bay of Biscay southwards to Lagos and in western Morocco (Fig. 4b), but during this period, kelp forests displayed a general decreasing trend, with a set of major contractions in the years 1990, 1996, 1998, 2000, 2004, 2007 and 2011 (Fig. 5a). While some of these were followed by putative recolonisations leading to no net change (e.g., 1996, 1998 and 2007), the years 1990, 2000, 2004 and 2011 were particularly severe for *S. polyschides*, producing permanent distribution losses of ~30%, when compared to a reference point of 13360 km^2^ for 1986 (Fig. 5a). These losses were most evident on the shores of northern Spain, southern Portugal and western Morocco (Fig. 4b), setting distributional limits similar to present ones (i.e., from Cabo de Peñas - ROI1 - to Carrapateira - ROI2 - and in western Morocco; Fig. 4a; 9766 km^2^ predicted area in 2014). The reconstruction of distributions predicted further range shifts in the near future. Using the Representative Concentration Pathway (RCP) 2.6, a scenario where greenhouse gas emissions are largely reduced over time, the losses were ~21% from 2014 onwards, with major breaks in 2017, 2036 and 2057 (Fig. 5a), restricting distributions to northwestern Iberia (Fig. 4c,d). The scenario RCP8.5, characterized by increasing gas emissions over time, predicted breaks in 2033, 2057, 2068 and from 2082 onwards (Fig. 5a). This scenario also restricts kelp forests to northwestern Iberia (Fig. 4e,f), although with losses of ~38% (6043 km^2^ in 2100) when compared to 2014. Despite more losses over the long term, the forecasts using RCP8.5 predicted a larger distribution in the first 19 years, leading to ~9% more kelp in RCP8.5 than RCP2.6 for the period 2015–2033 (8741 km^2^ ± 399 and 9619 km^2^ ± 398, respectively; Fig. 5a), spread between southwestern Portugal and western Morocco (Fig. 4c,e).

## Discussion

This study inferred the major environmental forces shaping the distribution of an important habitat forming species at its southern latitudinal range edge. It also predicted significant range losses during the recent past and for future scenarios of climate change, and demonstrated the feasibility of using a complex life cycle approach for the reconstruction of species’ distributions, along with the integration of potential dispersal and delayed development phases. It further allowed contradicting the general message associating range shifts with increasing temperature overpassing lethal limits^27^. By modelling distributions using environmental predictors with detailed spatial and temporal resolution (daily data), it allowed identifying novel effects that would be missed at a coarse scale. Namely, the years with recruitment failure and subsequent absence of adults were explained by: 1) the number of consecutive days with temperatures above the physiological tolerance of microscopic stages (i.e., gametophytes and young sporophytes), and 2) low nutrients during spring, associated with higher temperatures and poor upwelling. This study also showed evidence for temporal dispersal mediated by microscopic stages, which may maintain population sizes during unfavourable climate conditions. Such complexity of seasonal effects acting on different life history stages produced a more realistic simulation of inter-annual range shifting^28^ and would likely pass unnoticed with more simplistic modelling approaches.

Niche models corroborate empirical studies showing that the demography of this kelp is mainly determined at the gametophyte and young sporophyte stages during winter and spring^12^,^29^. In particular, the number of consecutive winter days with temperatures above 18 °C had the largest effect on the models’ response. During the winter season, fertilization of the female gametophyte is perhaps the most vulnerable phase of the life cycle of *S. polyschides*, having reduced fertility if exposed to temperatures above ~18 °C^25^. Extreme temperatures also determine the actual survival of gametophytes during this season, however with a higher critical threshold of 23 °C^30^. Such temperatures are not uncommon in winter throughout Iberia-Morocco, with some sites experiencing up to 129 days above 18 °C and maximum temperatures above 25 °C (Fig. 1). Whenever these thresholds are surpassed, the viability of microscopic stages, and therefore population sizes for following years, might be compromised and result in local extinctions, as those that have been documented by recent observations^6^,^21^,^23^,^25^.

Spring temperatures above 23 °C can also be detrimental for *S. polyschides*, particularly for the growth of young sporophytes^25^,^31^. However, our models discarded the effect of such extreme temperatures (average contribution <1%) and placed emphasis on the role of nutrients for microscopic stages to resume new cohorts^32^,^33^ (as shown for the southern Californian populations of *Macrocystis pyrifera*^34^). This was supported by the large effect of consecutive spring days with temperatures above 18 °C, the threshold at which the Iberia-Morocco region starts to be nutrient depleted (Nitrates and Phosphates; Supplementary Figure S2). The additional contribution of consecutive spring days with favourable upwelling conditions further emphasizes the central role of nutrients to the recruitment of *S. polyschides* at its southern range edge. In fact, the important role of nutrients for this species was previously suggested^35^, with nutrients rather than temperature explaining the overwintering of sporophytes in Brittany but not in Portugal. If recruitment fails due to unfavourable conditions, the continuity of population sizes can only rely on microscopic stages able to arrest development for additional periods of time.

The models further showed that compared to temperature and upwelling conditions, disturbance from waves play a small role in defining the range edge of S. polyschides, probably because waves primarily affect changes in biomass^11^,^36^ rather than explaining the complete loss of populations, as tested in the binomial models. Other variables not included in the models can further influence the demography of this species. For instance, low salinity (<9 PSS) and irradiance thresholds (<10–30 lux) inhibit the growth of *S. polyschides*^37^,^38^. However, the spatial scale of our models, composed by coastal cells of ~5 km, misses enough resolution to include the hyposaline regions of Iberia-Morocco (e.g., large estuaries and coastal lagoons) and to discriminate between coastal depths, therefore coastal cells containing presence data also include deeper areas where irradiance is limiting^39^. Photoperiodic responses such as winter irradiance limiting the development of new sporophytes^38^ could also be neglected in our modelling framework as these mechanisms mediate phenology only and not the inter-annual variability of recruitment.

The simulations testing different dispersal thresholds supported the hypothesis that *S. polyschides* has a limited degree of dispersal. The estimate of 5 km per year is in line with studies of *Macrocystis pyrifera* that showed dispersal in scales of a couple of kilometers^16^,^17^. In addition, this distance seems reasonable considering that most spores of the kelps *Macrocystis pyrifera* and *Pterygophora californica* stop swimming after ~24 h^40^. Despite this, one can’t discard the likely effect of large-scale dispersal mediated by floating rafts of kelp. These events certainly must have occurred when distant habitats such as offshore seamounts were firstly colonized by *S. polyschides*^6^,^7^, although they must be very uncommon since the spores released by rafts have a very low probability of reaching suitable rocky habitats in adequate densities to allow an effective colonization^17^, as fertilization is dependent upon the proximity of male and female gametophytes^41^.

The inferred latency period of microscopic stages was two years, meaning that populations of *S. polyschides* may be allowed to persist for longer than the natural cycle between two consecutive generations of sporophytes, a hypothesis that has been suggested by empirical records^6^. Most of what is known about the longevity of these stages is derived from laboratory experiments, with empirical evidence from culturing demonstrating that they last for several years^42^,^43^. Field evidence for the Iberian Peninsula demonstrated the existence of delayed development stages on, at least, the order of months^18^, and surveys performed in western Iberia^6^ revealed local extinctions followed by fast recolonizations that could be explained by delayed microscopic stages triggered by favourable environmental conditions (also hypothesised by other studies^25^). If these cryptic stages can actually persist for more than one year, as inferred by our simulations, they would allow the co-existence of multiple cohorts of this annual species and rescue populations whenever unfavourable conditions halt recruitment.

Compared to our study, niche modelling often focuses on a single life stage of a species (e.g., macroscopic sporophytes), neglecting the role of life stages with distinct traits^44^. By integrating the complete life-cycle of *S. polyschides*, together with spatial and temporal dispersal, the predictive capacity of our models was strongly improved (Supplementary Figure S3). This approach yielded excellent agreement^26^ between the predicted distribution and the actual distributional records (presences and absences), both for the full temporal span of hindcasts and outside the training window of the models (i.e., independent dataset prior to 2010; Fig. 3). Furthermore, because distributions were reconstructed from a starting point in the past, erroneous hindcasts should propagate until more recent times, which was not the case. The predictions for the present largely matched the known distribution of the species (as inferred from our field surveys and other studies^24^,^25^), with the only missing occurrences in Tarifa - Strait of Gibraltar. This is a particular region benefiting from unique environmental conditions allowing populations to persist in deep waters^7^,^39^. Specifically, the low light attenuation of Mediterranean waters allows sufficient irradiance at depths >25 m^7^,^39^, where the SST sensor OSTIA fails to detect suitable temperatures. Also, the permanent upwelling located in this region of the Gibraltar Strait provides favourable nutrient-rich conditions not found anywhere else throughout the surrounding areas^39^. Despite this, predictions largely matched the known distribution and allowed inferring the decline of ~30% of kelp forests from 1986 to 2014. The recent warming of sea surface waters is a well-documented trend for Iberia-Morocco^14^, and the predicted range shifts were coincident with the heat waves of 1988–1990, 1996–1998, 2003–2004 and 2006–2007^21^,^22^,^45^. These results highlight that climate change is paced by such discrete extreme warming episodes and range shifts often occur abruptly, as biological tipping-points are exceeded^46^. Some particular years showed poor recruitment (e.g., 1996–1998 and 2006–2007), however our results suggest that permanent losses occur mainly when unfavourable conditions arise on successive years, supporting the hypothesis that delayed stages may hold on populations sizes between consecutive generations of sporophytes.

The inferred distributional losses in northern Spain (eastwards to Cabo the Penãs) and southern Portugal (Lagos) are corroborated by our field surveys and additional studies^6^,^24^,^25^, which systematically fail to detect the species throughout these coastlines, despite its occurrence there in the past (Fig. 4a). These range shifts pushed the current distribution to the northwestern Iberian Peninsula, and discontinuous occurrences along southwestern Portugal and western Morocco, but only in upwelling regions^47^. There, cold and nutrient-rich upwelled waters form climatic refugia that buffer the overall warming trend observed in Iberia-Morocco^47^. It has been suggested that throughout the world upwelling is becoming more persistent, longer and stronger^48^. This may well be the case for southwestern Portugal and western Morocco^48^, where kelps prevail despite local extinctions and reduced abundances^6^. However, the seasonality and intensity of the northwest Iberian upwelling system decreased recently^49^. This was not linked to extinctions in our study, nor in other studies^25^, but throughout the extant populations of northwestern Iberia, individuals live fewer months and a smaller proportion became reproductive when compared to the populations studied there before the 2000s^25^.

The rapid range shifts predicted for *S. polyschides*, corroborated by our field evidence and other studies, suggest niche conservatism, which is not uncommon for marine species^50^. This suggests low plasticity or adaptive capacity to extreme climate conditions, as species that experienced warming-driven range shifts in the past are most likely to shift ranges when exposed to future warming events. Many species are predicted to shift ranges throughout the Iberia-Morocco region during the 21st century^8^,^9^. Our projections are in agreement with these expectations, however the use of different greenhouse gas emissions led to different predicted consequences. In the most optimistic scenario (RCP2.6), *S. polyschides* is predicted to decrease ~21% from 2014 to 2057, with extant populations confined to the refugium located on the northwest of the Iberia Peninsula. The predictions made with increasing emissions over time (RCP8.5) were more severe (as in other studies^8^,^9^), with losses of up to 38% by the end of the century. Such disparities between climate scenarios were only evident from 2068 onwards, and the more aggressive scenario predicted less loss in the first 19 years of forecasts. This is explained by the RCP8.5 data variability, which assumes more favourable winter conditions during 2017–2024 than the RCP2.6 (Supplementary Figure S4). This is plausible, as climate scenarios strongly diverge from 2050 onwards^51^. The RCP2.6 has a peak of radiative forcing (difference of energy absorbed and radiated by the Earth^51^) by 2050 followed by a decline until 2100^52^, whereas RCP8.5 represents the upper range in the literature, predicting higher radiative forcing by the end of the century^53^. However, one can’t discard that RCP8.5 may underestimate the warming in those initial years, as documented for the drying and cooling of Iberia-Morocco during the Last Glacial Maximum (20,000 years before present) in some AOGCM simulations^54^. If so, our models might over-predict distributions with RCP8.5, meaning that the loss of kelp forests could happen earlier and more severely, as some populations of southwest Portugal and western Morocco would not persist as predicted. Because forecasts had their starting point in the present, which was accurately predicted, the putative sources of bias are mostly rooted on the inherent uncertainties of climate simulations^51^.

In the future, dispersal might not be a suitable escape mechanism for populations to persist or recover along the Iberia-Morocco coastlines since there are important barriers halting migration. Our results estimate that the limited dispersal potential of *S. polyschides* (5 km per year) is not sufficient to connect and thereby recover the populations inhabiting the western continental edges of the Iberian Peninsula and Morocco, within their generational time frame. This inference is supported by genetic data that shows lack of interpopulation connectivity in that specific region^7^. The loss of most range edge marine forests of kelp seems plausible under future climate scenarios, particularly in the long term, and the consequences that will arise should be considered. Both scenarios used in this study suggest that this will occur for populations in the north and southwest portions of the Iberian Peninsula and throughout western Morocco. Such extinctions would erode unique genetic lineages^6^,^7^ thereby reducing the overall gene pool of this species and compromising its evolutionary potential^3^. In this context, deep offshore reefs may act as buffers to climate change, by allowing persistence of unique genetic groups in greater depths, away from the warmer surface trends^7^. This might be the case of the genetic diversity of western Morocco, that might prevail in one of such cryptic refugium (Gorringe offshore seamount), but no further evidence about a genetic refugial role of offshore deep sites has been found throughout the distributional range of *S. polyschides*^6^,^7^. Along side with genetic erosion, the demise of a main canopy-building species in the coastlines of Iberia-Morocco is expected to cause major changes in the whole ecosystem, particularly in the overall biomass and diversity of the numerous associated species^4^. Additionally, the loss of canopy cover releases important resources on the reef surface (e.g., light and space^55^) facilitating colonization by more warm-tolerant species^56^. This tends to lead to less structurally diverse habitats composed by crustose and foliose turf algae, characteristic of warmer or impacted and degraded ecosystems^57^. In the light of climate induced range shifts, our broad scale field surveys coupled with the predictions performed through time represent a unique baseline to reassess in the future the range edge distribution of Atlantic marine forests. This will allow the assessment of the hypotheses and predictions raised in this study, particularly whether range shifts for *S. polyschides* might not be rescued by dispersal, potentially driving major biodiversity loss and changes in ecosystem functioning.

## Methods

### Focal species and study region

The present study focused on the annual kelp *S. polyschides*, along the coastlines of the Iberian Peninsula and northern Africa, from the Bay of Biscay to southern Morocco, and throughout the Spanish, Moroccan and Algerian Mediterranean coasts (~5000 Km). This region is an appropriate model to address our questions because the populations of *S. polyschides* display unique phenology there^21^,^35^ and their ecological niche in this region may differ from that of populations inhabiting the centre of their distribution. Beyond Iberia-Morocco, seasonal *S. polyschides* only occurs on the offshore banks of Gorringe, Alboran and Messina^7^; these are particular deep habitats not affected by climate variability as shallow coastal waters, thus not useful for the goals of this study.

### Data on species occurrences and climate

Presence and absence records of *S. polyschides* were gathered by means of SCUBA diving and snorkelling in the summers of 2003, 2008, 2010, 2011 and 2012 (Supplementary Table S1). These surveys were performed on rocky reef sites by swimming randomly for periods up to 60 min. To avoid false absences, if no kelp was found within a site, at least one other survey was performed within 1 km from the initial one. Historical records (since 1986) were also collated from literature (Supplementary Table S1). New and historical data were gridded into 0.05° cells (~5 km) to match the resolution of the environmental predictors (see below).

Multiple seasonal environmental predictors were generated for modelling purposes (Supplementary Table S5). These predictors reflected factors putatively affecting the physiology of *S. polyschides* (Sea Surface Temperature - SST), disturbances that impact its habitat (Significant Wave Height - SWH) and essential resources (Coastal Upwelling Index - CUI - as a proxy for nutrients). Seasons were defined as “spring” (months = MAMJ), “winter” (months = ONDJF) and “previous summer” (months = JAS) to match the life cycle of this species and its unique phenology throughout Iberia-Morocco: the populations of *S. polyschides* in this region display a sharp recruitment of sporophytes in spring, followed by a peek of high abundances throughout summer; the adults release spores in late summer and die in the autumn; settled spores produce gametophytes that after fertilization persist until favourable spring conditions to resume recruitment^35^.

To hindcast distributions, daily data on SST and SWH were collated from the Operational Sea Surface Temperature and Sea Ice Analysis (OSTIA^58^) and the European Centre for Medium-Range Weather Forecasts (ECMWF^59^), respectively. Upwelling indices were produced with daily data from the ECMWF following a standardized implementation^60^. This approach uses the eastward and northward components of surface wind speed to estimate Ekman’s offshore transport of water masses (m^3^/s). Deep nutrient rich waters are expected to start flowing toward the surface at Coastal Upwelling Index (CUI) >16 m^3^/s^61^. To forecast distributions, SST and wind speed data were collated on a daily basis from two Atmospheric-Ocean General Circulation Models (AOGCM): the Community Climate System Model (CCSM4) and the Model for Interdisciplinary Research on Climate (MIROC5). This was performed under two Representative Concentration Pathways (RCP^51^): the RCP2.6, where greenhouse gas emissions are largely reduced over time, and the RCP8.5, characterized by increasing gas emissions over time. Because SWH data were not available on AOGCMs, forecasts did not consider this variable. However, this limitation was likely not important to model results because the range edge of *S. polyschides* was largely explained by SST and CUI alone (see Results). All environmental predictors were gridded to a common resolution of 0.05° using bilinear interpolation.

### Ecological Niche Modeling

The ENM approach used followed the methods of other studies, which take advantage of a cross-validation procedure to capture the most transferrable (i.e., temporal and/or spatial cross-applicability) and parsimonious set of environmental predictors among numerous candidates^7^,^8^,^9^. To this end, we adopted Boosted Regression Trees (BRT^62^), a machine-learning method with the ability to fit non-linear relationships with complex interactions among predictors.

Because *S. polyschides* may be absent from some sites due to factors other than niche availability (e.g., dispersal limitations), the BRT models were developed with pseudo-absences outside the species’ ecological space rather than the absences recorded in field surveys^7^,^8^,^9^. This information was obtained from a habitat suitability surface produced with Mahalanobis distance, which related the normalized environmental predictors with the presence records^63^. The same amount of pseudo-absences as the presence records were extracted from cells with probability scores less than or equal to 0.2^64^. These were randomly chosen from environmentally dissimilar cells identified with K-means clustering on the normalized predictors, and using the number of presence records as the k clustering parameter^65^.

Cross-validation was performed non-randomly on temporally distinct datasets, by iteratively training models in a given year and testing predictive performances on another year (as largely recommend^66^). The years used were 2010 and 2012 because these stand for the most complete surveys (Supplementary Table S1). All combinations of environmental predictors showing no signs of strong correlation (Spearman’s R < |0.8|; Supplementary Figure S5) were used to train BRT models. This allowed reducing over-parameterization, which can narrow niche estimates, and provided a basis to discuss the individual effect of predictors^67^. Each single model using BRT is dependent on a set of parameters (number of trees; learning rate, tree complexity and bag fraction), which when carefully identified increase the level of generality and reduce overfitting^62^. These parameters were determined by testing a range of number of trees (100 to 10000, step 50), learning rates (0.01, 0.005, 0.001) and tree complexities (from 1 up to the total number of predictors in a model) in a 10-fold cross-validation performed in the training dataset, using lowest deviance as a measure of success^7^,^8^,^9^,^62^. A bag fraction of 0.5 (i.e., the proportion of data used at each iteration) was used as default value because it produces optimal binomial responses^62^. To further reduce the potential for overfitting, predictors were forced to produce monotonic responses, negative (for SST and SWH) or positive (for CUI).

The potential of each combination of predictors for transferability was verified using the information of presence records only^68^. To this end, the Minimum Predicted Area criterion (MPA) was used to measure the proportion of predicted area with suitable habitat in the test dataset^69^, when applying a threshold retrieving at least 0.9 sensitivity. MPA ranged from zero to one, with lower values producing smaller predicted areas. Accordingly, the models with higher potential for transferability were identified as those producing the lowest MPA in the cross-validation iterations^69^. The relative contribution of each predictor to the distribution of *S. polyschides* was assessed by determining the mean reduction in MPA when adding a given predictor to all alternative models. In this step, the mean MPA scores were transformed as 1 - MPA and rescaled to sum 100, so that higher relative scores would stand for stronger contributions.

### The range edge of *S. polyschides* through time

The suitable habitats of *S. polyschides* in Iberia-Morocco (probability of occurrence) were predicted by merging (mean function) the responses of the most transferable models, when assigned on a yearly basis to the environment conditions of the period 1986–2100. Because the models only fitted non-correlated predictors, it was possible to discard predictors with lower predictive performances among highly correlated pairs. Since ensemble modelling is also useful for uncertain datasets, forecasts merged the different outcomes from CCSM4 and MIROC5^7^,^8^,^9^.

To reconstruct the distribution of this species through time, the suitable habitats were converted to binary responses (presence or absence) based on a MPA threshold. The accuracy of predicted distributions was assessed with TSS using all distributional records (including absences) both in space and time. In this process, we tested three different types of reconstructions: (1) no dispersal, with cells experiencing permanent absence in time whenever the habitat becomes unsuitable, (2) unlimited dispersal, with presences and absences as a direct function of habitat suitability, and (3) specific estimates of potential dispersal distance and arrested development (latency) of microscopic stages. Since there is no robust information about these metrics, we simulated their effect on the marginal score of TSS. Multiple reconstructions of the distribution were generated with increasing dispersal distances (D; 1 to 500 km, step 1 km) and latency periods (L; 1 to 5 years, step 1 year), starting from a state of no dispersal and no latency. Because no reference point was available about past distributions, the simulations started with the earliest predicted distribution (year 1986). From this state on, for every *i* cell predicted with suitable habitat at a given *t* year, a presence in *i* at *t* was assigned if one of the following conditions were met: (1) there was a previous presence in *i* cell at any *t* − L year; (2) there was a rescue effect from neighbouring cells with presence records at *t* − 1 year, distancing equal or less D km from *i*. Distances between pairs of cells were determined using the shortest path along the coast. The simulations retrieved 2500 matrices, and the best combination of D and L was found as the one retrieving the highest marginal value of TSS. The implementation of these thresholds will be propagated in the reconstruction of this species’ distribution through time. While spatial dispersal (D) allows the (re) colonization of formerly unsuitable habitats, temporal dispersal (L; i.e., delayed stages) enables local persistence whenever unsuitable habitats arise.

The area covered by kelp along the Iberia-Morocco coastline was estimated through time. The final accuracy of predictions was inferred with TSS, sensitivity and specificity using all distributional records (i.e., 1986–2014) and independently per year. This latter approach provides an independent framework to evaluate the accuracy of the hindcasting, particularly when considering the historical records outside the training window of the models (i.e., independent dataset prior to 2010). Persistence, extinction and extinctions followed by recolonizations were mapped for the periods 1986–2014, 2015–2050 and 2051–2100.

All analyses described were conducted in R^70^ using packages adehabitat, dismo, doParallel, gbm, geoR, ncdf4, parallel, raster, rgdal, sp, SDMTools.

## Additional Information

**How to cite this article:** Assis, J. *et al*. Major shifts at the range edge of marine forests: the combined effects of climate changes and limited dispersal. *Sci. Rep.* **7**, 44348; doi: 10.1038/srep44348 (2017).

**Publisher's note:** Springer Nature remains neutral with regard to jurisdictional claims in published maps and institutional affiliations.

## Supplementary Material

Supporting Information

## References

[b1] Chen I. C., Hill J. K., Ohlemüller R., Roy D. B. & Thomas C. D. Rapid range shifts of species associated with high levels of climate warming. Science 333, 1024–1026 (2011). 21852500 10.1126/science.1206432

[b2] Bellard C., Bertelsmeier C., Leadley P., Thuiller W. & Courchamp F. Impacts of climate change on the future of biodiversity. Ecol. Lett. 15, 365–377 (2012). 22257223 10.1111/j.1461-0248.2011.01736.x PMC3880584

[b3] Hampe A. & Petit R. J. Conserving biodiversity under climate change: The rear edge matters. Ecol. Lett. 8, 461–467 (2005). 21352449 10.1111/j.1461-0248.2005.00739.x

[b4] Hoegh-Guldberg O. & Bruno J. F. The impact of climate change on the world’s marine ecosystems. Science 328, 1523–1528 (2010). 20558709 10.1126/science.1189930

[b5] Steneck R. S. . Kelp forest ecosystems: biodiversity, stability, resilience and future. Environ. Conserv. 29, 436–459 (2002).

[b6] Assis J. . High and distinct range-edge genetic diversity despite local bottlenecks. PLoS One 8, e68646 (2013). 23967038 10.1371/journal.pone.0068646 PMC3744244

[b7] Assis J. . Deep reefs are climatic refugia for genetic diversity of marine forests. J. Biogeogr. 43, 833–844 (2016).

[b8] Assis J., Lucas A. V., Bárbara I. & Serrão E. A. Future climate change is predicted to shift long-term persistence zones in the cold-temperate kelp *Laminaria hyperborea*. Mar. Environ. Res. 113, 174–182 (2016). 26608411 10.1016/j.marenvres.2015.11.005

[b9] Neiva J. . Genes left behind: Climate change threatens cryptic genetic diversity in the canopy-forming seaweed *Bifurcaria bifurcata*. PLoS One 10, e0131530 (2015). 26177545 10.1371/journal.pone.0131530 PMC4503591

[b10] Guisan A. & Thuiller W. Predicting species distribution: Offering more than simple habitat models. Ecol. Lett. 8, 993–1009 (2005). 10.1111/j.1461-0248.2005.00792.x 34517687

[b11] Cavanaugh K. C., Siegel D. A., Reed D. C. & Dennison P. E. Environmental controls of giant-kelp biomass in the Santa Barbara Channel, California. Mar. Ecol. Prog. Ser. 429, 1–17 (2011).

[b12] Norton T. A. The factors influencing the distribution of *Saccorhiza polyschides* in the region of Lough Ine. J. Mar. Biol. Assoc. United Kingdom 58, 527–536 (1978).

[b13] Taboada A., von Wehrden H. & Assmann T. Integrating Life Stages into Ecological Niche Models: A Case Study on Tiger Beetles. PLoS One 8, e70038 (2013). 23894582 10.1371/journal.pone.0070038 PMC3720956

[b14] Lima F. P. & Wethey D. S. Three decades of high-resolution coastal sea surface temperatures reveal more than warming. Nat. Commun. 3, 1–13 (2012). 10.1038/ncomms1713 22426225

[b15] Arribas P. . Evaluating drivers of vulnerability to climate change: A guide for insect conservation strategies. Glob. Chang. Biol. 18, 2135–2146 (2012).

[b16] Gaylord B., Reed D. C. D. C., Raimondi P. T. & Washburn L. Macroalgal spore dispersal in coastal environments: mechanistic insights revealed by theory and experiment. Ecol. Monogr. 76, 481–502 (2006).

[b17] Reed D. D. C. . A metapopulation perspective on the patch dynamics and connectivity of giant kelp In Marine Metapopulations (eds Kritzer J. P. & Sale F. P.). 353–386 (San Diego, 2006).

[b18] Barradas A., Alberto F., Engelen A. H. & Serrão E. A. Fast sporophyte replacement after removal suggests banks of latent microscopic stages of *Laminaria Ochroleuca* (phaeophyceae) in tide pools in northern Portugal. Cah. Biol. Mar. 52, 435–439 (2011).

[b19] Horta E Costa B. . Tropicalization of fish assemblages in temperate biogeographic transition zones. Mar. Ecol. Prog. Ser. 504, 241–252 (2014).

[b20] Belkin I. M. Rapid warming of large marine ecosystems. Prog. Oceanogr. 81, 207–213 (2009).

[b21] Voerman S. E., Llera E. & Rico J. M. Climate driven changes in subtidal kelp forest communities in NW Spain. Mar. Environ. Res. 90, 119–127 (2013). 23948150 10.1016/j.marenvres.2013.06.006

[b22] Kersting D. K., Bensoussan N. & Linares C. Long-term responses of the endemic reef-builder *Cladocora caespitosa* to Mediterranean warming. PLoS One 8, e70820 (2013). 23951016 10.1371/journal.pone.0070820 PMC3741371

[b23] Nicastro K. R. . Shift happens: trailing edge contraction associated with recent warming trends threatens a distinct genetic lineage in the marine macroalga *Fucus vesiculosus*. BMC Biol. 11, 6 (2013). 23342999 10.1186/1741-7007-11-6 PMC3598678

[b24] Araújo R. M. . Status, trends and drivers of kelp forests in Europe: an expert assessment. Biodivers. Conserv. 25, 1319–1348 (2016).

[b25] Fernandez C. The retreat of large brown seaweeds on the north coast of Spain: the case of *Saccorhiza polyschides*. Eur. J. Phycol. 46, 352–360 (2011).

[b26] Allouche O., Tsoar A. & Kadmon R. Assessing the accuracy of species distribution models: Prevalence, kappa and the true skill statistic (TSS). J. Appl. Ecol. 43, 1223–1232 (2006).

[b27] Burrows M. T. . Geographical limits to species-range shifts are suggested by climate velocity. Nature 507, 492–5 (2014). 24509712 10.1038/nature12976

[b28] Takao S. . An improved estimation of the poleward expansion of coral habitats based on the inter-annual variation of sea surface temperatures. Coral Reefs 34, 1125–1137 (2015).

[b29] den Hoek C. & Hoek C. Van Den. The distribution of benthic marine algae in relation to the temperature regulation of their life histories. Biol. J. Linn. Soc. 18, 81–144 (1982).

[b30] tom Dieck I. T. Temperature tolerance and survival in darkness of kelp gametophytes (*Laminariales*, Phaeophyta) - Ecological and biogeographical implications. Mar. Ecol. Prog. Ser. 100, 253–264 (1993).

[b31] Norton T. A. Experiments on the factors influencing the geographical distributions of *Saccorhiza polyschides* and *Saccorhiza dermatodea*. New Phytol. 78, 625–635 (1977).

[b32] Carney L. T. & Edwards M. S. Role of nutrient fluctuations and delayed development in gametophyte reproduction by *Macrocystis pyrifera* (phaeophyceae) in Southern California. J. Phycol. 46, 987–996 (2010).

[b33] Lewis R. J., Green M. K. & Afzal M. E. Effects of chelated iron on oogenesis and vegetative growth of kelp gametophytes (Phaeophyceae). Phycol. Res. 61, 46–51 (2013).

[b34] Dayton P. K., Tegner M. J., Edwards P. B. & Riser K. L. Temporal and spatial scales of kelp demography: The role of oceanographic climate. Ecol. Monogr. 69, 219–250 (1999).

[b35] Pereira T. R., Engelen A. H., Pearson G. A., Valero M. & Serrão E. A. Contrasting timing of life stages across latitudes – a case study of a marine forest-forming species. Eur. J. Phycol. 50, 361–369 (2015).

[b36] Cavanaugh K. C. . Synchrony in dynamics of giant kelp forests is driven by both local recruitment and regional environmental controls. Ecology 94, 499–509 (2013). 23691668 10.1890/12-0268.1

[b37] Norton T. A. Synopsis of biological data on *Saccorhiza polyschides*. FAO Fisheries Synopsis 83, 11–93.

[b38] Norton T. A. & Burrows E. M. The environmental control of the seasonal development of Saccorhiza polyschides. Proc. Int. Seaweed Symp. 6, 287–296 (1969).

[b39] Flores-Moya A. In Seaweed Biology: Novel Insights into Ecophysiology, Ecology and Utilization (eds. Wiencke C. & Bischof K.) 315–327 (Springer Berlin Heidelberg, 2012).

[b40] Reed D. C., Amsler C. D. & Ebeling A. W. Dispersal in kelps: factors affecting spore swimming and competency. Ecology 73, 1577–1585 (1992).

[b41] Reed D. C., Neushul M. & Ebeling A. W. The role of density on gametophyte growth and reproduction in the kelps *Macrocystis pyrifera* and *Pterygophora californica*. J. Phycol 27, 361–366 (1991).

[b42] Neushul M. In The effects of waste disposal on kelp communities (ed. Univ Calif Inst Mar Res) 282–300 (Santa Barbara, 1983).

[b43] Kornmann P. & Sahling P. H. Kalkbohrende Mikrothalli bei Helminthocladia and Scinaia (Nemaliales, Rhodophyta). Helgol Meeresunters 34, 31–40 (1980).

[b44] Taboada A., von Wehrden H. & Assmann T. Integrating Life Stages into Ecological Niche Models: A Case Study on Tiger Beetles. PLoS One 8, (2013). 10.1371/journal.pone.0070038 PMC3720956 23894582

[b45] García-Herrera R., Díaz J., Trigo R. M., Luterbacher J. & Fischer E. M. A review of the European Summer heat wave of 2003. Crit. Rev. Environ. Sci. Technol. 40, 267–306 (2010).

[b46] Harley C. D. G. & Paine R. T. Contingencies and compounded rare perturbations dictate sudden distributional shifts during periods of gradual climate change. Proc. Natl. Acad. Sci. USA 106, 11172–11176 (2009). 19541649 10.1073/pnas.0904946106 PMC2708713

[b47] Lourenço C. R. . Upwelling areas as climate change refugia for the distribution and genetic diversity of a marine macroalga. J. Biogeogr. 43, 1595–1607 (2016).

[b48] McGregor H. V., Dima M., Fischer H. W. & Mulitza S. Rapid 20th-century increase in coastal upwelling off northwest Africa. Science 315, 637–639 (2007). 17272719 10.1126/science.1134839

[b49] Alvarez I. . Comparative analysis of upwelling influence between the western and northern coast of the Iberian Peninsula. Cont. Shelf Res. 31, 388–399 (2011).

[b50] Peterson A. T. Ecological niche conservatism: A time-structured review of evidence. J. Biogeogr. 38, 817–827 (2011).

[b51] Moss R. H. . The next generation of scenarios for climate change research and assessment. Nature 463, 747–756 (2010). 20148028 10.1038/nature08823

[b52] van Vuuren D. P. . RCP2.6: Exploring the possibility to keep global mean temperature increase below 2 °C. Clim. Change 109, 95–116 (2011).

[b53] Riahi K. . RCP 8.5-A scenario of comparatively high greenhouse gas emissions. Clim. Change 109, 33–57 (2011).

[b54] Ramstein G. . How cold was Europe at the Last Glacial Maximum? A synthesis of the progress achieved since the first PMIP model-data comparison. Clim. Past Discuss. 3, 197–220 (2007).

[b55] Gorman D. & Connell S. D. Recovering subtidal forests in human-dominated landscapes. J. Appl. Ecol. 46, 1258–1265 (2009).

[b56] Lima F. P., Ribeiro P. A., Queiroz N., Hawkins S. J. & Santos A. M. Do distributional shifts of northern and southern species of algae match the warming pattern? Glob. Chang. Biol. 13, 2592–2604 (2007).

[b57] Wernberg T. . An extreme climatic event alters marine ecosystem structure in a global biodiversity hotspot. *Nat*. Clim. Chang. 3, 78–82 (2012).

[b58] Donlon C. J. . The Operational Sea Surface Temperature and Sea Ice Analysis (OSTIA) system. Remote Sens. Environ. 116, 140–158 (2012).

[b59] Dee D. P. . The ERA-Interim reanalysis: Configuration and performance of the data assimilation system. Q. J. R. Meteorol. Soc. 137, 553–597 (2011).

[b60] Perez B. Comparison of upwelling indices off Baja California derived from three different wind data sources. CalCOFl Rep 48, 204– 214 (2007).

[b61] Alvarez I., Gomez-Gesteira M., deCastro M., Gomez-Gesteira J. L. & Dias J. M. Summer upwelling frequency along the western Cantabrian coast from 1967 to 2007. J. Mar. Syst. 79, 218–226 (2010).

[b62] De’ath G. Boosted trees for ecological modeling and prediction. Ecology 88, 243–251 (2007). 17489472 10.1890/0012-9658(2007)88[243:btfema]2.0.co;2

[b63] Calenge C., Darmon G., Basille M., Loison a. & Jullien J. M. The factorial decomposition of the Mahalanobis distances in habitat selection studies. Ecology 89, 555–566 (2008). 18409444 10.1890/06-1750.1

[b64] Chefaoui R. M. & Lobo J. M. Assessing the effects of pseudo-absences on predictive distribution model performance. Ecol. Modell. 210, 478–486 (2008).

[b65] Senay S. D., Worner S. P. & Ikeda T. Novel three-step pseudo-absence selection technique for improved species distribution modelling. PLoS One 8, e71218 (2013). 23967167 10.1371/journal.pone.0071218 PMC3742778

[b66] Wenger S. J. & Olden J. D. Assessing transferability of ecological models: An underappreciated aspect of statistical validation. Methods Ecol. Evol. 3, 260–267 (2012).

[b67] Peterson A. T. T. . Ecological niches and geographic distributions. Choice Reviews Online 49, (Princeton University Press, 2011).

[b68] Lobo J. M., Jiménez-valverde A. & Real R. AUC: A misleading measure of the performance of predictive distribution models. Glob. Ecol. Biogeogr. 17, 145–151 (2008).

[b69] Franklin J. Mapping species distributions. (Cambridge University Press, 2010).

[b70] R Team. R Development Core Team. R A Lang. Environ. Stat. Comput. 55, 275–286 (2013).

