# Supplementary material for: Major shifts at the range edge of marine forests: the combined effects of climate changes and limited dispersal
Source: Sci Rep. 2017 Mar 9;7:44348. doi: 10.1038/srep44348 (PMC5343584; doi:10.1038/srep44348)

Supplementary Information

Major shifts at the range edge of marine forests: the combined effects of climate changes and limited dispersal

Assis, J.*[1], Berecibar, E.[2], Claro, B.[1], Alberto, F.[3], Reed, D.[4], Raimondi, P.[5], Serrão, E.A.[1]

[1] CCMAR, Centro de Ciências do Mar, Universidade do Algarve, Campus de Gambelas, 8005-139 Faro, Portugal.

[2] Estrutura de Missão para a Extensão da Plataforma Continental (EMEPC), Rua Costa Pinto 165, 2770-042, Paço de Arcos, Portugal

[3] Department of Biological Sciences, University of Wisconsin-Milwaukee, Milwaukee, Wisconsin 53201 USA

[4] Marine Science Institute, University of California, Santa Barbara, California 93106 USA

[5] Department of Biology, University of California, Santa Cruz, California 95064 USA

Corresponding author (*): Jorge Assis (jorgemfa@gmail.com | +351 912 361 127)

Supplementary Figure S1. Study region (~5000 km). Orange contour along the coast represent the modelling cells on a 5 km resolution. (Left panel) Sampling effort for *Saccorhiza polyschides* (year; black dots represent sampling sites). (Right panel) Data compiled from literature (black dots represent sites where information for *S. polyschides* was available). Maps generated with QGIS (QGIS Development Team, 2016. QGIS Geographic Information System. Open Source Geospatial Foundation Project. http://www.qgis.org/).

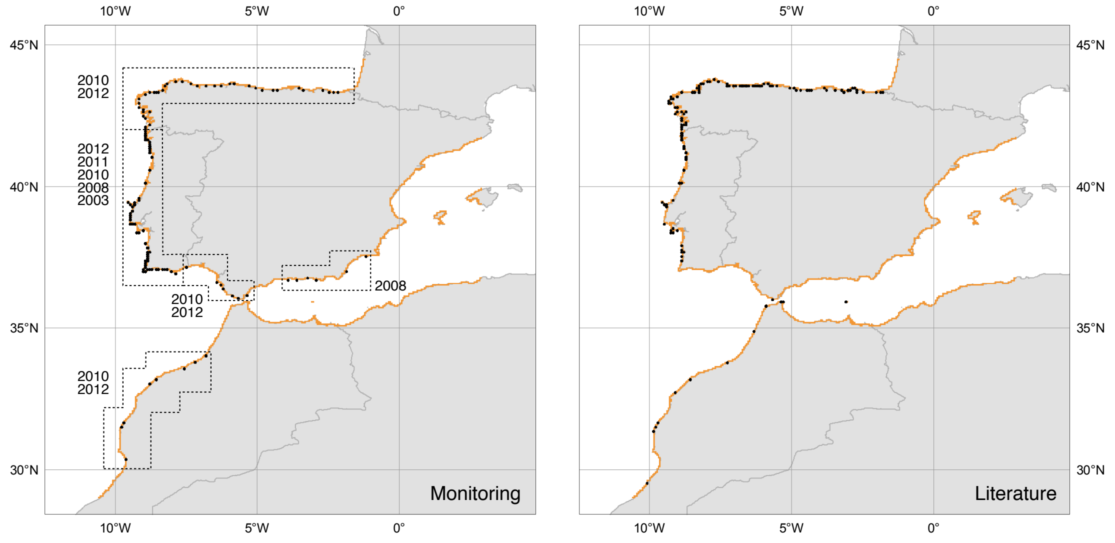

Supplementary Table S1. Records of occurrence used for modelling the distribution of *Saccorhiza polyschides* with information for Longitude (Lon), Latitude (Lat), Year, presence or absence (PA), type of record (Field survey or literature) and Reference citation.

Lon	Lat	Year	PA	Type	Reference
-8.875	41.775	2003	1	Monitoring	-
-8.875	41.675	2003	1	Monitoring	-
-8.775	41.475	2003	1	Monitoring	-
-8.725	41.275	2003	1	Monitoring	-
-8.725	41.225	2003	1	Monitoring	-
-8.675	41.025	2003	1	Monitoring	-
-8.775	40.625	2003	1	Monitoring	-
-8.875	40.175	2003	1	Monitoring	-
-9.125	39.525	2003	1	Monitoring	-
-9.325	39.375	2003	1	Monitoring	-
-9.475	38.875	2003	1	Monitoring	-
-9.475	38.725	2003	1	Monitoring	-
-9.325	38.675	2003	1	Monitoring	-
-8.975	38.475	2003	1	Monitoring	-
-9.225	38.425	2003	1	Monitoring	-
-9.125	38.425	2003	1	Monitoring	-
-8.875	37.975	2003	1	Monitoring	-
-8.925	37.225	2003	1	Monitoring	-
-8.525	37.125	2003	0	Monitoring	-
-8.675	37.075	2003	1	Monitoring	-
-8.475	37.075	2003	0	Monitoring	-
-8.225	37.075	2003	0	Monitoring	-
-8.925	37.025	2003	1	Monitoring	-
-8.025	37.025	2003	0	Monitoring	-
-6.425	36.675	2003	0	Monitoring	-
-8.875	41.825	2008	1	Monitoring	-
-8.875	41.675	2008	1	Monitoring	-
-8.825	41.625	2008	1	Monitoring	-
-8.775	41.425	2008	1	Monitoring	-
-8.775	41.375	2008	1	Monitoring	-
-8.725	41.175	2008	1	Monitoring	-
-8.675	41.025	2008	1	Monitoring	-
-8.875	40.175	2008	1	Monitoring	-
-9.325	39.375	2008	1	Monitoring	-
-9.375	39.125	2008	1	Monitoring	-
-9.425	38.975	2008	1	Monitoring	-
-9.225	38.425	2008	1	Monitoring	-
-8.825	37.625	2008	1	Monitoring	-
-1.175	37.525	2008	0	Monitoring	-
-8.825	37.425	2008	0	Monitoring	-
-7.475	37.175	2008	0	Monitoring	-
-8.525	37.125	2008	0	Monitoring	-
-8.975	37.075	2008	0	Monitoring	-
-8.775	37.075	2008	0	Monitoring	-
-8.175	37.075	2008	0	Monitoring	-
-6.725	37.075	2008	0	Monitoring	-

Lon	Lat	Year	PA	Type	Reference
-8.925	37.025	2008	0	Monitoring	-
-6.625	37.025	2008	0	Monitoring	-
-1.875	37.025	2008	0	Monitoring	-
-6.425	36.875	2008	0	Monitoring	-
-6.425	36.775	2008	0	Monitoring	-
-3.225	36.775	2008	0	Monitoring	-
-3.925	36.725	2008	0	Monitoring	-
-3.625	36.725	2008	0	Monitoring	-
-2.875	36.725	2008	0	Monitoring	-
-6.425	36.675	2008	0	Monitoring	-
-6.175	36.375	2008	0	Monitoring	-
-5.775	43.625	2010	1	Monitoring	-
-8.725	43.325	2010	1	Monitoring	-
-8.525	43.325	2010	1	Monitoring	-
-9.125	42.825	2010	1	Monitoring	-
-9.025	42.675	2010	1	Monitoring	-
-8.875	42.025	2010	1	Monitoring	-
-8.875	41.925	2010	1	Monitoring	-
-8.875	41.825	2010	1	Monitoring	-
-8.875	41.725	2010	1	Monitoring	-
-8.825	41.625	2010	1	Monitoring	-
-8.775	41.575	2010	1	Monitoring	-
-8.775	41.525	2010	1	Monitoring	-
-8.775	41.375	2010	1	Monitoring	-
-8.725	41.225	2010	1	Monitoring	-
-8.675	41.025	2010	1	Monitoring	-
-8.775	40.625	2010	1	Monitoring	-
-8.875	40.175	2010	1	Monitoring	-
-9.275	39.425	2010	1	Monitoring	-
-9.375	39.325	2010	1	Monitoring	-
-9.375	39.125	2010	1	Monitoring	-
-9.425	38.975	2010	1	Monitoring	-
-9.175	38.425	2010	1	Monitoring	-
-8.875	37.975	2010	0	Monitoring	-
-8.825	37.825	2010	0	Monitoring	-
-8.775	37.725	2010	0	Monitoring	-
-8.925	37.175	2010	1	Monitoring	-
-7.475	37.175	2010	0	Monitoring	-
-8.575	37.125	2010	0	Monitoring	-
-8.775	37.075	2010	0	Monitoring	-
-8.675	37.075	2010	0	Monitoring	-
-8.475	37.075	2010	0	Monitoring	-
-6.725	37.075	2010	0	Monitoring	-
-8.975	37.025	2010	0	Monitoring	-
-8.925	37.025	2010	0	Monitoring	-
-8.875	37.025	2010	0	Monitoring	-
-6.425	36.875	2010	0	Monitoring	-
-6.425	36.675	2010	0	Monitoring	-
-6.175	36.375	2010	0	Monitoring	-
-5.625	36.025	2010	1	Monitoring	-
-6.175	35.175	2010	0	Monitoring	-
-6.825	34.025	2010	0	Monitoring	-
-7.175	33.825	2010	0	Monitoring	-
-7.525	33.625	2010	0	Monitoring	-
-8.575	33.225	2010	1	Monitoring	-

Lon	Lat	Year	PA	Type	Reference
-8.725	33.025	2010	1	Monitoring	-
-9.675	31.725	2010	1	Monitoring	-
-9.775	31.525	2010	1	Monitoring	-
-9.625	30.375	2010	0	Monitoring	-
-8.875	41.825	2011	1	Monitoring	-
-8.875	41.675	2011	1	Monitoring	-
-8.825	41.625	2011	1	Monitoring	-
-8.775	41.425	2011	1	Monitoring	-
-8.725	41.175	2011	1	Monitoring	-
-8.675	41.025	2011	1	Monitoring	-
-8.875	40.175	2011	1	Monitoring	-
-9.325	39.375	2011	1	Monitoring	-
-9.375	39.125	2011	1	Monitoring	-
-9.425	38.975	2011	1	Monitoring	-
-9.225	38.425	2011	1	Monitoring	-
-9.125	38.425	2011	1	Monitoring	-
-8.875	37.975	2011	0	Monitoring	-
-8.875	37.325	2011	1	Monitoring	-
-8.775	37.075	2011	0	Monitoring	-
-8.675	37.075	2011	0	Monitoring	-
-8.475	37.075	2011	0	Monitoring	-
-8.275	37.075	2011	0	Monitoring	-
-8.125	37.075	2011	0	Monitoring	-
-8.975	37.025	2011	0	Monitoring	-
-8.925	37.025	2011	0	Monitoring	-
-8.025	37.025	2011	0	Monitoring	-
-7.825	36.975	2011	0	Monitoring	-
-5.625	36.025	2011	1	Monitoring	-
-6.175	35.175	2011	0	Monitoring	-
-6.825	34.025	2011	0	Monitoring	-
-8.575	33.225	2011	1	Monitoring	-
-9.675	31.725	2011	1	Monitoring	-
-9.625	30.375	2011	0	Monitoring	-
-7.875	43.725	2012	1	Monitoring	-
-7.625	43.725	2012	1	Monitoring	-
-8.125	43.675	2012	1	Monitoring	-
-7.325	43.625	2012	1	Monitoring	-
-5.925	43.625	2012	1	Monitoring	-
-5.775	43.625	2012	0	Monitoring	-
-8.225	43.575	2012	1	Monitoring	-
-7.075	43.575	2012	1	Monitoring	-
-6.825	43.575	2012	1	Monitoring	-
-6.475	43.575	2012	1	Monitoring	-
-6.275	43.575	2012	1	Monitoring	-
-5.275	43.525	2012	0	Monitoring	-
-5.025	43.475	2012	0	Monitoring	-
-3.525	43.475	2012	0	Monitoring	-
-8.275	43.425	2012	1	Monitoring	-
-4.775	43.425	2012	0	Monitoring	-
-4.425	43.425	2012	0	Monitoring	-
-4.275	43.425	2012	0	Monitoring	-
-3.325	43.425	2012	0	Monitoring	-
-2.675	43.425	2012	0	Monitoring	-
-8.425	43.375	2012	1	Monitoring	-
-8.625	43.325	2012	1	Monitoring	-

Lon	Lat	Year	PA	Type	Reference
-2.425	43.325	2012	0	Monitoring	-
-2.275	43.325	2012	0	Monitoring	-
-2.125	43.325	2012	0	Monitoring	-
-8.925	43.275	2012	1	Monitoring	-
-9.175	43.125	2012	1	Monitoring	-
-9.125	42.925	2012	1	Monitoring	-
-9.025	42.725	2012	1	Monitoring	-
-8.775	42.625	2012	1	Monitoring	-
-9.025	42.525	2012	1	Monitoring	-
-8.875	42.425	2012	1	Monitoring	-
-8.775	42.225	2012	1	Monitoring	-
-8.875	42.125	2012	1	Monitoring	-
-8.875	41.975	2012	1	Monitoring	-
-8.875	41.775	2012	1	Monitoring	-
-8.825	41.625	2012	1	Monitoring	-
-8.775	41.475	2012	1	Monitoring	-
-8.725	41.325	2012	1	Monitoring	-
-8.675	41.025	2012	1	Monitoring	-
-8.775	40.625	2012	1	Monitoring	-
-8.875	40.175	2012	1	Monitoring	-
-9.375	39.325	2012	0	Monitoring	-
-9.425	39.075	2012	1	Monitoring	-
-9.225	38.425	2012	1	Monitoring	-
-8.825	37.475	2012	1	Monitoring	-
-8.875	37.375	2012	1	Monitoring	-
-8.925	37.175	2012	1	Monitoring	-
-8.825	37.075	2012	0	Monitoring	-
-8.675	37.075	2012	0	Monitoring	-
-8.275	37.075	2012	0	Monitoring	-
-8.125	37.075	2012	0	Monitoring	-
-8.975	37.025	2012	0	Monitoring	-
-8.925	37.025	2012	0	Monitoring	-
-7.825	36.975	2012	0	Monitoring	-
-5.625	36.025	2012	1	Monitoring	-
-6.825	34.025	2012	0	Monitoring	-
-7.175	33.825	2012	0	Monitoring	-
-7.525	33.625	2012	0	Monitoring	-
-8.725	33.025	2012	1	Monitoring	-
-9.675	31.725	2012	1	Monitoring	-
-9.775	31.525	2012	1	Monitoring	-
-9.625	30.375	2012	0	Monitoring	-
-5.775	43.625	1986	1	Literature	1
-5.725	43.575	1986	1	Literature	1
-8.375	43.375	1986	1	Literature	1
-3.175	43.375	1986	1	Literature	2
-8.875	42.425	1986	1	Literature	1
-8.175	43.625	1987	1	Literature	1
-3.175	43.375	1987	1	Literature	1
-9.025	42.675	1987	1	Literature	1
-7.275	43.575	1988	1	Literature	1
-6.025	43.575	1988	1	Literature	1
-4.275	43.425	1988	1	Literature	1
-2.925	43.425	1988	1	Literature	1
-3.125	43.375	1988	1	Literature	1
-2.675	43.375	1988	1	Literature	1

Lon	Lat	Year	PA	Type	Reference
-1.825	43.375	1988	1	Literature	1
-1.775	43.375	1988	1	Literature	1
-1.975	43.325	1988	1	Literature	1
-9.025	43.225	1988	1	Literature	1
-9.225	43.175	1988	1	Literature	1
-8.725	42.675	1988	1	Literature	1
-8.225	43.425	1989	1	Literature	1
-3.775	43.425	1989	1	Literature	1
-8.825	43.375	1989	1	Literature	1
-8.375	43.375	1989	1	Literature	1
-8.275	43.375	1989	1	Literature	1
-9.075	42.775	1989	1	Literature	1
-8.975	42.775	1989	1	Literature	1
-8.875	42.425	1989	1	Literature	1
-8.825	41.675	1989	1	Literature	1
-8.675	41.175	1989	1	Literature	1
-8.875	40.175	1989	1	Literature	1
-9.125	39.525	1989	1	Literature	1
-9.475	38.725	1989	1	Literature	1
-8.975	38.475	1989	1	Literature	1
-5.275	35.925	1989	1	Literature	3
-5.575	35.825	1989	1	Literature	3
-8.575	33.225	1989	1	Literature	3
-8.175	43.625	1990	1	Literature	1
-6.675	43.575	1990	1	Literature	1
-6.625	43.575	1990	1	Literature	1
-6.375	43.575	1990	1	Literature	1
-8.425	43.375	1990	1	Literature	1
-9.225	43.125	1990	1	Literature	1
-9.175	43.125	1990	1	Literature	1
-9.025	42.675	1990	1	Literature	1
-8.725	42.675	1990	1	Literature	1
-8.775	41.425	1990	1	Literature	1
-9.525	39.425	1990	1	Literature	4
-7.275	43.625	1991	1	Literature	1
-7.225	43.575	1991	1	Literature	1
-3.825	43.475	1991	1	Literature	1
-2.825	43.475	1991	1	Literature	5
-2.775	43.475	1991	1	Literature	5
-3.225	43.425	1991	1	Literature	5
-2.975	43.425	1991	1	Literature	5
-8.225	43.375	1991	1	Literature	1
-8.825	43.325	1991	1	Literature	1
-2.425	43.325	1991	1	Literature	5
-9.275	42.975	1991	1	Literature	1
-8.775	42.575	1991	1	Literature	1
-8.325	43.475	1992	1	Literature	1
-4.875	43.475	1992	1	Literature	1
-4.475	43.425	1992	1	Literature	1
-8.425	43.375	1992	1	Literature	1
-8.825	42.175	1992	1	Literature	1
-8.775	42.175	1992	1	Literature	1
-6.525	43.575	1993	1	Literature	1
-6.175	43.575	1993	1	Literature	1
-7.025	43.525	1993	1	Literature	1

Lon	Lat	Year	PA	Type	Reference
-8.875	42.125	1993	1	Literature	1
-8.875	42.075	1993	1	Literature	1
-8.875	41.925	1993	1	Literature	1
-8.875	41.775	1993	1	Literature	1
-8.875	41.725	1993	1	Literature	1
-9.225	38.425	1993	1	Literature	6
-8.825	37.925	1993	1	Literature	7
-8.825	37.775	1993	1	Literature	7
-8.775	37.725	1993	1	Literature	7
-8.825	37.675	1993	1	Literature	7
-8.825	37.525	1993	1	Literature	7
-8.825	37.425	1993	1	Literature	7
-8.825	37.375	1993	1	Literature	7
-5.625	36.025	1993	1	Literature	1
-6.575	43.575	1994	1	Literature	1
-8.725	42.225	1994	1	Literature	1
-5.375	35.925	1995	1	Literature	8
-5.875	35.825	1995	1	Literature	8
-5.575	35.825	1995	1	Literature	8
-5.875	35.775	1995	1	Literature	8
-9.675	31.725	1995	1	Literature	3
-9.175	38.525	1996	1	Literature	9
-9.225	38.475	1996	1	Literature	9
-9.225	38.425	1996	1	Literature	9
-9.175	38.425	1996	1	Literature	9
-9.125	38.425	1996	1	Literature	9
-5.375	35.925	1996	1	Literature	8
-5.875	35.825	1996	1	Literature	8
-5.575	35.825	1996	1	Literature	8
-5.875	35.775	1996	1	Literature	8
-9.675	31.725	1996	1	Literature	3
-9.225	38.425	1997	1	Literature	10
-8.875	41.875	1999	1	Literature	11
-8.875	41.775	1999	1	Literature	11
-8.825	41.625	1999	1	Literature	11
-8.775	41.475	1999	1	Literature	11
-8.775	41.375	1999	1	Literature	11
-8.725	41.225	1999	1	Literature	11
-8.675	41.075	1999	1	Literature	11
-8.675	40.975	1999	1	Literature	11
-8.775	40.625	1999	1	Literature	11
-8.925	40.175	1999	1	Literature	11
-8.875	41.875	2000	1	Literature	11
-8.875	41.775	2000	1	Literature	11
-8.825	41.625	2000	1	Literature	11
-8.775	41.475	2000	1	Literature	11
-8.775	41.375	2000	1	Literature	11
-8.725	41.225	2000	1	Literature	11
-8.675	41.075	2000	1	Literature	11
-8.675	40.975	2000	1	Literature	11
-8.775	40.625	2000	1	Literature	11
-8.925	40.175	2000	1	Literature	11
-9.225	38.425	2000	1	Literature	9
-8.875	41.875	2001	1	Literature	11
-8.875	41.775	2001	1	Literature	11

Lon	Lat	Year	PA	Type	Reference
-8.825	41.625	2001	1	Literature	11
-8.775	41.475	2001	1	Literature	11
-8.775	41.375	2001	1	Literature	11
-8.725	41.225	2001	1	Literature	11
-8.675	41.075	2001	1	Literature	11
-8.675	40.975	2001	1	Literature	11
-8.775	40.625	2001	1	Literature	11
-8.925	40.175	2001	1	Literature	11
-8.875	41.875	2002	1	Literature	11
-8.875	41.775	2002	1	Literature	11
-8.825	41.625	2002	1	Literature	11
-8.775	41.475	2002	1	Literature	11
-8.775	41.375	2002	1	Literature	11
-8.725	41.225	2002	1	Literature	11
-8.675	41.075	2002	1	Literature	11
-8.675	40.975	2002	1	Literature	11
-8.775	40.625	2002	1	Literature	11
-8.925	40.175	2002	1	Literature	11
-8.575	33.225	2002	1	Literature	3
-9.775	31.525	2002	1	Literature	3
-8.875	41.875	2003	1	Literature	11
-8.875	41.775	2003	1	Literature	11
-8.825	41.625	2003	1	Literature	11
-8.775	41.475	2003	1	Literature	11
-8.775	41.375	2003	1	Literature	11
-8.725	41.225	2003	1	Literature	11
-8.675	41.075	2003	1	Literature	11
-8.675	40.975	2003	1	Literature	11
-8.775	40.625	2003	1	Literature	11
-8.925	40.175	2003	1	Literature	11
-3.175	43.375	2003	0	Literature	12
-8.875	41.875	2004	1	Literature	11
-8.875	41.775	2004	1	Literature	11
-8.825	41.625	2004	1	Literature	11
-8.775	41.475	2004	1	Literature	11
-8.775	41.375	2004	1	Literature	11
-8.725	41.225	2004	1	Literature	11
-8.675	41.075	2004	1	Literature	11
-8.675	40.975	2004	1	Literature	11
-8.775	40.625	2004	1	Literature	11
-8.925	40.175	2004	1	Literature	11
-8.875	41.875	2005	1	Literature	11
-8.875	41.775	2005	1	Literature	11
-8.825	41.625	2005	1	Literature	11
-8.775	41.475	2005	1	Literature	11
-8.775	41.375	2005	1	Literature	11
-8.725	41.225	2005	1	Literature	11
-8.675	41.075	2005	1	Literature	11
-8.675	40.975	2005	1	Literature	11
-8.775	40.625	2005	1	Literature	11
-8.925	40.175	2005	1	Literature	11
-2.975	43.425	2006	1	Literature	12
-8.875	41.875	2006	1	Literature	11
-8.875	41.775	2006	1	Literature	11
-8.825	41.625	2006	1	Literature	11

Lon	Lat	Year	PA	Type	Reference
-8.775	41.475	2006	1	Literature	11
-8.775	41.375	2006	1	Literature	11
-8.725	41.225	2006	1	Literature	11
-8.675	41.075	2006	1	Literature	11
-8.675	40.975	2006	1	Literature	11
-8.775	40.625	2006	1	Literature	11
-8.925	40.175	2006	1	Literature	11
-2.675	43.425	2007	1	Literature	12
-8.875	41.875	2007	1	Literature	11
-8.875	41.775	2007	1	Literature	11
-8.825	41.625	2007	1	Literature	11
-8.775	41.475	2007	1	Literature	11
-8.775	41.375	2007	1	Literature	11
-8.725	41.225	2007	1	Literature	11
-8.675	41.075	2007	1	Literature	11
-9.425	39.375	2007	1	Literature	13
-9.375	39.375	2007	1	Literature	13
-3.175	43.375	2007	0	Literature	12
-2.675	43.425	2008	1	Literature	12
-9.425	39.375	2008	1	Literature	5
-2.825	43.475	2008	0	Literature	5
-2.775	43.475	2008	0	Literature	5
-3.225	43.425	2008	0	Literature	5
-2.975	43.425	2008	0	Literature	5
-3.175	43.375	2008	0	Literature	12
-2.425	43.325	2008	0	Literature	5
-3.425	43.425	2009	1	Literature	14
-7.725	43.775	2010	1	Literature	15
-7.925	43.725	2010	1	Literature	15
-7.875	43.725	2010	1	Literature	15
-7.575	43.725	2010	1	Literature	15
-8.075	43.675	2010	1	Literature	15
-5.975	43.625	2010	1	Literature	15
-8.275	43.575	2010	1	Literature	15
-7.175	43.575	2010	1	Literature	15
-6.975	43.575	2010	1	Literature	15
-6.825	43.575	2010	1	Literature	15
-6.675	43.575	2010	1	Literature	15
-6.475	43.575	2010	1	Literature	15
-6.275	43.575	2010	1	Literature	15
-6.075	43.575	2010	1	Literature	15
-8.275	43.475	2010	1	Literature	15
-8.375	43.375	2010	1	Literature	15
-8.875	43.325	2010	1	Literature	15
-8.975	43.225	2010	1	Literature	15
-9.175	43.125	2010	1	Literature	15
-9.275	43.025	2010	1	Literature	15
-9.225	42.925	2010	1	Literature	15
-9.025	42.775	2010	1	Literature	15
-8.875	42.625	2010	1	Literature	15
-9.075	42.575	2010	1	Literature	15
-8.875	42.425	2010	1	Literature	15
-8.875	42.325	2010	1	Literature	15
-8.825	42.325	2010	1	Literature	15
-8.725	42.275	2010	1	Literature	15

Lon	Lat	Year	PA	Type	Reference
-8.825	42.125	2010	1	Literature	15
-5.325	35.925	2010	1	Literature	16
-3.025	35.925	2010	1	Literature	16
-5.775	43.625	2010	0	Literature	15
-5.675	43.575	2010	0	Literature	15
-5.475	43.575	2010	0	Literature	15
-5.025	43.475	2010	0	Literature	15
-4.875	43.475	2010	0	Literature	15
-4.775	43.425	2010	0	Literature	15
-4.625	43.425	2010	0	Literature	15
-4.475	43.425	2010	0	Literature	15
-4.375	43.425	2010	0	Literature	15
-8.725	41.175	2012	1	Literature	3
-9.375	39.325	2012	1	Literature	3
-5.325	35.925	2012	1	Literature	16
-3.025	35.925	2012	1	Literature	16
-9.075	32.725	2012	1	Literature	3
-9.825	31.375	2012	1	Literature	3
-5.975	43.625	2007	1	Literature	15
-6.975	43.575	2007	1	Literature	15
-6.825	43.575	2007	1	Literature	15
-6.675	43.575	2007	1	Literature	15
-6.475	43.575	2007	1	Literature	15
-6.275	43.575	2007	1	Literature	15
-6.075	43.575	2007	1	Literature	15
-5.881	43.630	2007	1	Literature	15
-6.152	43.569	2007	1	Literature	15
-6.562	43.575	2007	1	Literature	15
-5.775	43.625	2007	0	Literature	15
-5.675	43.575	2007	0	Literature	15
-5.475	43.575	2007	0	Literature	15
-5.025	43.475	2007	0	Literature	15
-4.875	43.475	2007	0	Literature	15
-4.775	43.425	2007	0	Literature	15
-4.625	43.425	2007	0	Literature	15
-4.475	43.425	2007	0	Literature	15
-4.375	43.425	2007	0	Literature	15
-5.161	43.492	2007	0	Literature	15
-5.975	43.625	2008	1	Literature	15
-6.975	43.575	2008	1	Literature	15
-6.825	43.575	2008	1	Literature	15
-6.675	43.575	2008	1	Literature	15
-6.475	43.575	2008	1	Literature	15
-6.275	43.575	2008	1	Literature	15
-6.075	43.575	2008	1	Literature	15
-5.881	43.630	2008	1	Literature	15
-6.152	43.569	2008	1	Literature	15
-6.562	43.575	2008	1	Literature	15
-5.775	43.625	2008	0	Literature	15
-5.675	43.575	2008	0	Literature	15
-5.475	43.575	2008	0	Literature	15
-5.025	43.475	2008	0	Literature	15
-4.875	43.475	2008	0	Literature	15
-4.775	43.425	2008	0	Literature	15
-4.625	43.425	2008	0	Literature	15

Lon	Lat	Year	PA	Type	Reference
-4.475	43.425	2008	0	Literature	15
-4.375	43.425	2008	0	Literature	15
-5.161	43.492	2008	0	Literature	15
-5.975	43.625	2009	1	Literature	15
-6.975	43.575	2009	1	Literature	15
-6.825	43.575	2009	1	Literature	15
-6.675	43.575	2009	1	Literature	15
-6.475	43.575	2009	1	Literature	15
-6.275	43.575	2009	1	Literature	15
-6.075	43.575	2009	1	Literature	15
-5.881	43.630	2009	1	Literature	15
-6.152	43.569	2009	1	Literature	15
-6.562	43.575	2009	1	Literature	15
-5.775	43.625	2009	0	Literature	15
-5.675	43.575	2009	0	Literature	15
-5.475	43.575	2009	0	Literature	15
-5.025	43.475	2009	0	Literature	15
-4.875	43.475	2009	0	Literature	15
-4.775	43.425	2009	0	Literature	15
-4.625	43.425	2009	0	Literature	15
-4.475	43.425	2009	0	Literature	15
-4.375	43.425	2009	0	Literature	15
-5.161	43.492	2009	0	Literature	15

References

1. Izquierdo, J. L., Gallardo, T. & Pérez-Ruzafa, I. M. Mapas de distribución de algas marinas de la Península Ibérica e Islas Baleares. IX. *Saccorhiza polyschides* (Lightf.) Batt. y *Chorda filum* (l.) Stackh. (*Laminariales*, *Fucophyceae*). *Bot. Complut*. **20**, 105–115 (1995).

2. Borja A. & Gorostiaga, J.M. Distribucion geografica de *Saccorhiza polyschides* (Ligthf.) Batt. en la costa vasca. Su posible relacion con la temperatura. *Bentos* **6**, 1–8 (1990).

3. Benhissoune, S., Boudouresque, C. F. & Verlaque, M. A check-list of marine seaweeds of the Mediterranean and Atlantic coasts of Morocco. I. *Chlorophyceae* wille s. l. *Bot. Mar*. **44**, 171–182 (2001).

4. Pereira, L. et al. *MACOI, Portuguese Seaweeds Website. World-wide Electronic Publication. IMAR, Department of Life Sciences*, University of Coimbra (2014) Available at: http://macoi.ci.uc.pt. (Accessed: 1th May 2015).

5. Díez, I., Muguerza, N., Santolaria, A., Ganzedo, U. & Gorostiaga, J. M. Seaweed assemblage changes in the eastern Cantabrian Sea and their potential relationship to climate change. *Estuar. Coast. Shelf Sci.* **99**, 108–120 (2012).

6. Santos, R. A multivariate study of biotic and abiotic relationships in a subtidal algal stand. *Mar. Ecol. Prog. Ser*. **94**, 181–190 (1993).

7. Carvalho, M. A. D. S. A. *Estudo das comunidades intertidais de macroalgas bentónicas da Área de Paisagem Protegida do Sudoeste Alentejano e Costa Vicentina*. (e.d. Universidade do Algarve) (Faro, 1993).

8. Riadi H., Kazzaz, M. & Ater, M. Contribution a La Connaissance Des Algues Marines Benthiques Du Detroit De Gibraltar Et De La Mediterranee Occidentale Marocaine *Chlorophyceae* Et *Phaeophyceae*. *Acta. Bot. Malacit*. **25**, 5-20 (2000).

9. Almada, V., Gonçalves, E. & Henriques, M. *Inventariação e ecologia da ictiofauna do substrato rochoso da costa Arrábida/Espichel. Instuto Superior de Psicologia Aplicada / Instituto da Conservação da Natureza*. Technical report. (Lisboa, 2002).

10. Henriques, M. & Almada, V. C. Relative importance of cleaning behavior in *Centrolabrus exoletus* and other wrasse at Arrábida, Portugal. *J. Mar. Biol. Ass*. **77**, 891–898 (1997).

11. Araújo, R. et al. Checklist of benthic marine algae and cyanobacteria of northern Portugal. *Bot. Mar.* **52**, 24–46 (2009).

12. Alcock, R. *The Effects of Climate Change on Rocky Shore Communities in the Bay of Biscay*, 1895-2050. PhD Dissertation (ed. University of Southampton) (United Kingdom, 2003).

13. Antunes, D. M. T., Quintino, V. M. S. & Rodrigues, A. M. J. *Identificação e caracterização dos biótopos de uma praia de Peniche*. Technical report. (ed. Universidade de Aveiro) (Aveiro, 2008).

14. González-Irusta, J.M. Contribución al conocimiento del erizo de mar Paracentrotus lividus, (Lamarck, 1816) en el Mar Cantábrico: ciclo gonadal y dinámica de poblaciones. PhD Dissertation (ed. Universidad de Cantabria) (Santander, 2009).

15. Fernandez C. The retreat of large brown seaweeds on the north coast of Spain: the case of *Saccorhiza polyschides*. *Eur. J. Phycol*. **46**, 352–360 (2011).

16. Aguilar, R. & Garcia-Ubero, R. *Distribution of Deep-Sea Laminarians around three spanish Marine Protected Areas*. Technical report. (Oceana. Madrid, 2010).

Supplementary Table S2. Environmental predictors used for modelling the distribution of *Saccorhiza polyschides*. Name of predictors (SST - Sea Surface Temperature; SWH - Significant Wave Height; CUI – Coastal Upwelling Index; Winter: NDJF; Spring: MAMJ; Summer: JAS), range in the study region, units, rationale and mean relative contribution to the accuracy of models (bold values show predictors included in the final ensemble).

**Name**	**Range (unit)**	**Rationale**	**Contribution (%)**
Consecutive summer days with SST above 18°C	37-92 (days)	Can reduce the performance and growth of adult sporophytes[1,2,3]	9.25±0.76
Consecutive summer days with SST above 23°C	0-92 (days)	Can negatively affect the survival of sporophytes and the spore release, settlement and germination[1,3,4]	4.94±0.58
Maximum summer SST	19.17-29.28 (ºC)	Tipping point for the survival of sporophytes[1,5]	8.81±0.82
Consecutive winter days with SST above 18°C	0-129 (days)	Can negatively affect the fertility of the female gametophyte[3,4]	**15.82±0.82**
Consecutive winter days with SST above 23°C	0-28 (days)	Can negatively affect the survival of gametophytes and young microscopic sporophytes[3,6]	0.32±0.21
Maximum winter SST	17.88-25.54 (ºC)	Tipping point for the survival of gametophytes[3,6]	**13.61±0.82**
Consecutive spring days with SST above 18°C	0-85 (days)	Can reduce the performance and growth of young sporophytes[1,2,3,4]	**15.72±0.85**
Consecutive spring days with SST above 23°C	0-22 (days)	Can halt the growth and affect the survival of young sporophytes[1,3,4]	0.17±0.27
Maximum spring SST	17.46-27.02 (ºC)	Tipping point for the survival of sporophytes during rectruitment[1,3,5]	13.24±0.81
Consecutive summer days with SWH above 3m	0-7 (days)	Days of high waves can have a major negative effect on sporophytes[7,8]	-0.1±0.29
Maximum SWH of summer	1.57-7.48 (m)	Individual days of high waves can wipe out sporophytes[8,9]	**0.39±0.35**
Consecutive winter days with SWH above 3m	0-29 (days)	The development of gametophytes can be compromised due to scouring and burial[10,11]	-0.2±0.36
Maximum SWH of winter	2.75-10.53 (m)	Individual days of high waves can promote scouring and burial of gametophytes[10,11]	0.22±0.32
Consecutive spring days with SWH above 3m	0-12 (days)	The development of sporophytes can be compromised due to scouring and burial[10,11]	**0.25±0.38**
Maximum SWH of spring	2.32-9.20 (m)	Individual days of high waves can wipe out sporophytes during rectruitment[8,9]	-0.3±0.36
Consecutive summer days with CUI above 16	0-92 (days)	Favourable upwelling conditions driving nutrients for sporophytes[12,13]	3.28±0.48
Consecutive summer days with CUI above 32	0-82 (days)	Strong upwelling conditions driving nutrients for sporophytes[12,13]	2.20±0.49
Consecutive spring days with CUI above 16	2-110 (days)	Favourable upwelling conditions driving nutrients for sporophytes during rectruitment[12,13]	**8.44±0.47**
Consecutive spring days with CUI above 32	0-65 (days)	Strong upwelling conditions driving nutrients for sporophytes during rectruitment[12,13]	3.25±0.47

References

1. Norton, T. A. Experiments on the factors influencing the geographical distributions of *Saccorhiza polyschides* and *Saccorhiza dermatodea*. New Phytol. **78**, 625–635 (1977).

2. Wernberg, T. *et al*. Decreasing resilience of kelp beds along a latitudinal temperature gradient: Potential implications for a warmer future. *Ecol. Lett*. **13**, 685–694 (2010).

3. Hoek, C. Van Den. The distribution of benthic marine algae in relation to the temperature regulation of their life histories. *Biol. J. Linn. Soc*. **18**, 81–144 (1982).

4. Fernandez, C. The retreat of large brown seaweeds on the north coast of Spain: the case of *Saccorhiza polyschides*. *Eur. J. Phycol*. **46**, 352–360 (2011).

5. Lüning, K. Critical levels of light and temperature regulating the gametogenesis of 3 *Laminaria spp phaeophyceae*. *J. Phycol*. **16**, 1–15 (1980).

6. Dieck, I. T. Temperature tolerance and survival in darkness of kelp gametophytes (*Laminariales*, Phaeophyta) - Ecological and biogeographical implications. *Mar. Ecol. Prog. Ser*. **100**, 253–264 (1993).

7. Graham, M. H. *et al*. Population dynamics of giant kelp *Macrocystis pyrifera* along a wave exposure gradient. *Mar. Ecol. Prog. Ser*. **148**, 269–279 (1997).

8. Cavanaugh, K. C. *et al*. Synchrony in dynamics of giant kelp forests is driven by both local recruitment and regional environmental controls. *Ecology* **94**, 499–509 (2013).

9. Reed, D. C., Rassweiler, A. & Arkema, K. K. Biomass rather than growth rate determines variation in net primary production by giant kelp. *Ecology* **89**, 2493–2505 (2008).

10. Engledow, H. R. & Bolton, J. J. Seaweed alpha-diversity within the lower eulittoral zone in Namibia - the effects of wave action, sand inundation, mussels and limpets. *Bot. Mar*. **37**, 267–276 (1994).

11. Norton, T. A. The factors influencing the distribution of *Saccorhiza polyschides* in the region of Lough Ine. *J. Mar. Biol. Assoc*. **58**, 527–536 (1978).

12. Perez, B. Comparison of upwelling indices off Baja California derived from three different wind data sources. *CalCOFI Rep.* **48**, 204 – 214 (2007).

13. Alvarez, I., Gomez-Gesteira, M., deCastro, M., Gomez-Gesteira, J. L. & Dias, J. M. Summer upwelling frequency along the western Cantabrian coast from 1967 to 2007. *J. Mar. Syst*. **79**, 218–226 (2010).

Supplementary Figure S2. Relationship between temperature and nutrients (top left panel: Nitrates; top right panel: Phosphates) and between coastal upwelling index (averaged for spring; MAMJ) and nutrients (bottom left panel: Nitrates; bottom right panel: Phosphates) inferred for the study region. In situ data obtained from the World Ocean Atlas[1], CARbon IN the Atlantic[2] and the Global Ocean Data Analysis Project[3].

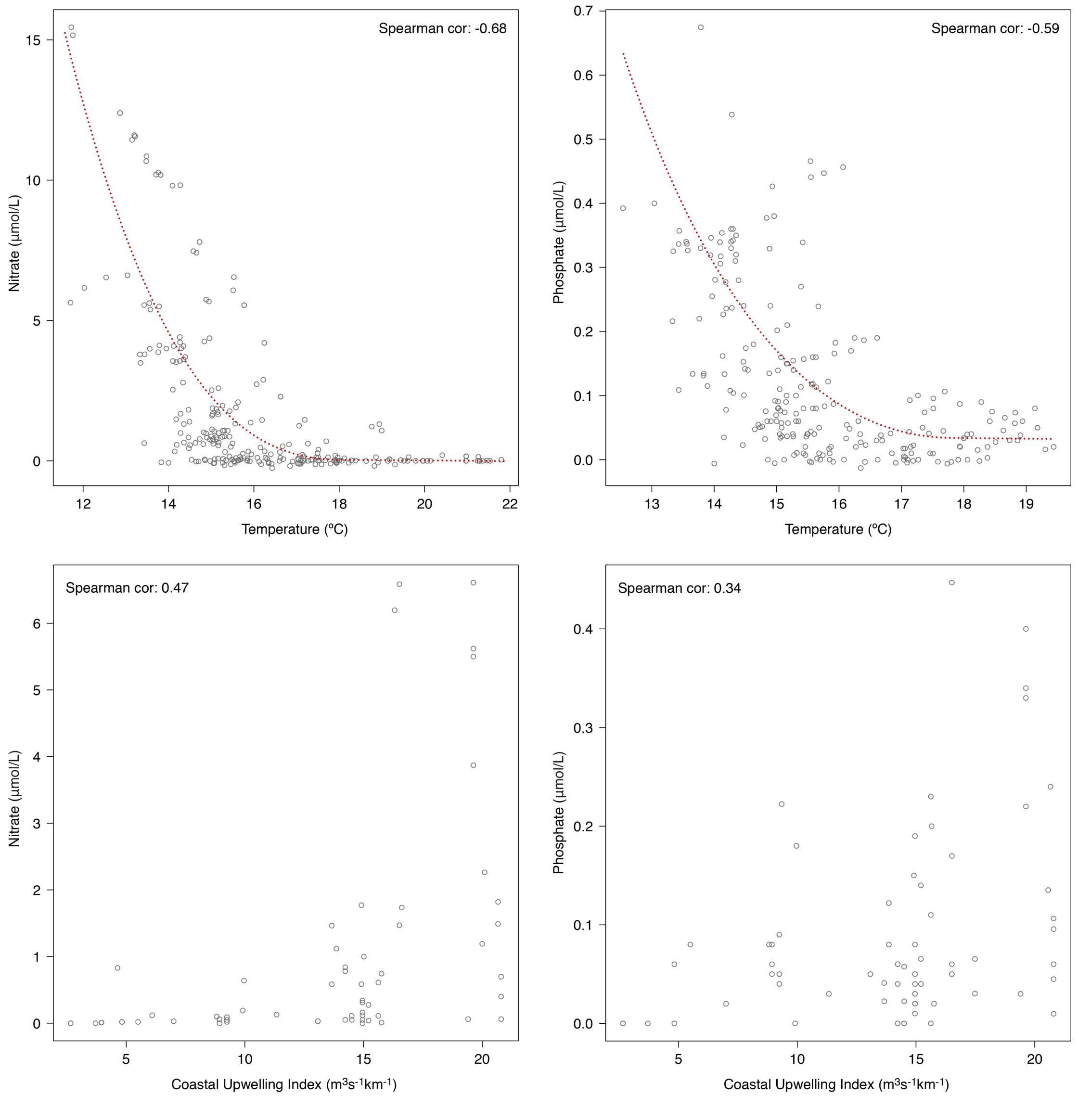

References

1. Locarnini, R. A., A. V. Mishonov, J. I. Antonov, T. P. Boyer, H. E. Garcia, O. K. Baranova, M. M. Zweng, C. R. Paver, J. R. Reagan, D. R. Johnson, M. Hamilton, and D. Seidov, 2013. World Ocean Atlas 2013, Volume 1: Temperature. S. Levitus, Ed., A. Mishonov Technical Ed.; NOAA Atlas NESDIS 73, 40 pp.

2. Tanhua, T., Brown, P. J., and Key, R. M.: CARINA: nutrient data in the Atlantic Ocean, Earth Syst. Sci. Data, 1, 7-24, 2009.

3. Key, R.M., A. Kozyr, C.L. Sabine, K. Lee, R. Wanninkhof, J. Bullister, R.A. Feely, F. Millero, C. Mordy, T.-H. Peng. 2004. A global ocean carbon climatology: Results from GLODAP. Global Biogeochemical Cycles, Vol. 18, GB4031 (PDF).

Supplementary Figure S3. Reconstruction of the species' distribution based on summer predictors only and no potential for temporal dispersal (macroscopic traits only). (Top left panel) Predicted distribution for 2014 and (right top panel) areas of persistence and extinction predicted from 1986 to 2014. (Bottom panel) Extent of *S. polyschides* in Iberia-Morocco predicted from 1986 to 2100 with remote sensing data and AOGCMs (under two scenarios of greenhouse gas emissions; RCP26 and RCP8.5). The inset displays the accuracy of hindcasts as True Skill Statistics, sensitivity (true presence rate) and specificity (true absence rate). Maps generated with QGIS (QGIS Development Team, 2016. QGIS Geographic Information System. Open Source Geospatial Foundation Project. http://www.qgis.org/).

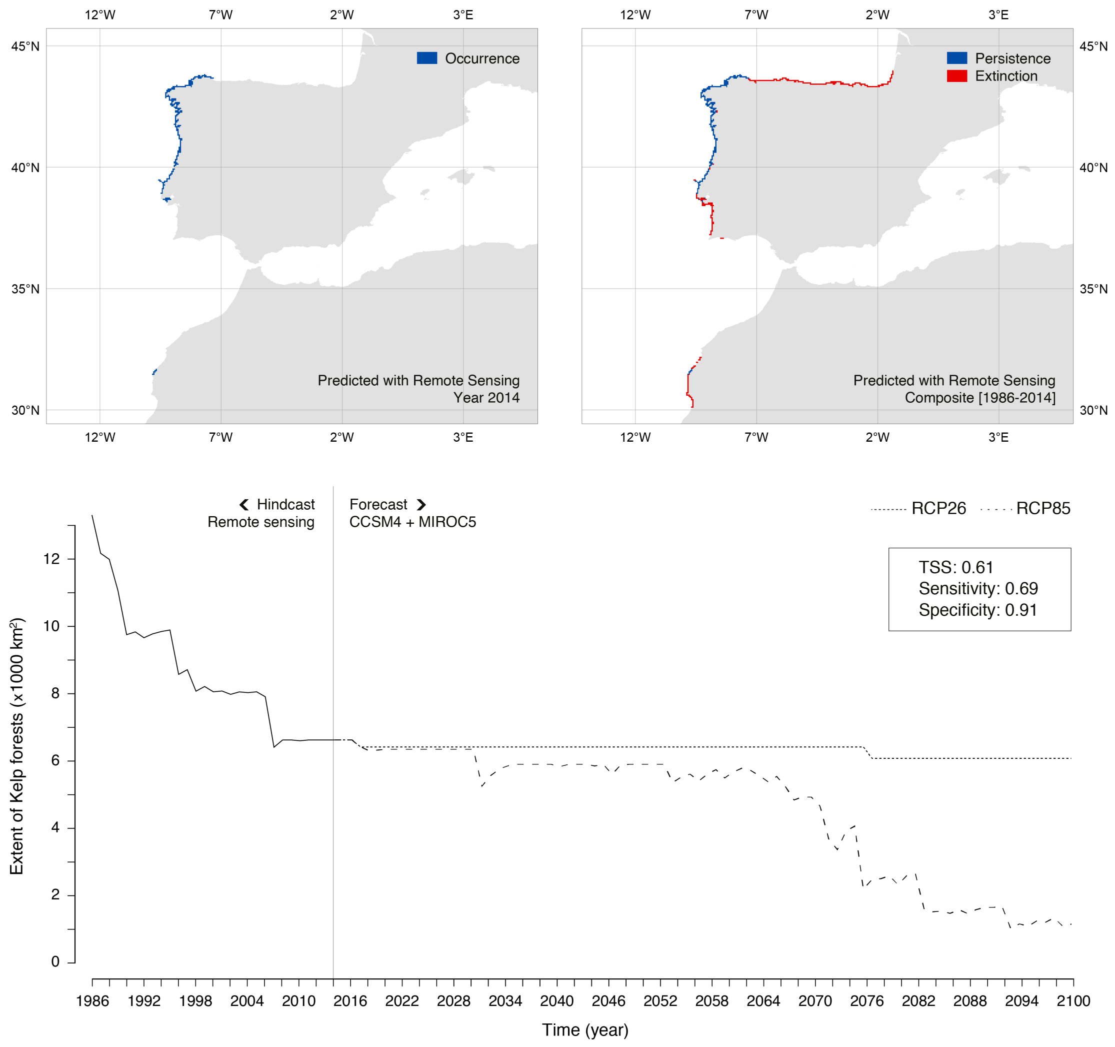

Supplementary Figure S4. Time-series of the most important predictors included in the final ensemble, averaged for the Iberia-Morocco region (SST - Sea Surface Temperature; CUI – Coastal Upwelling Index; Winter: NDJF; Spring: MAMJ).

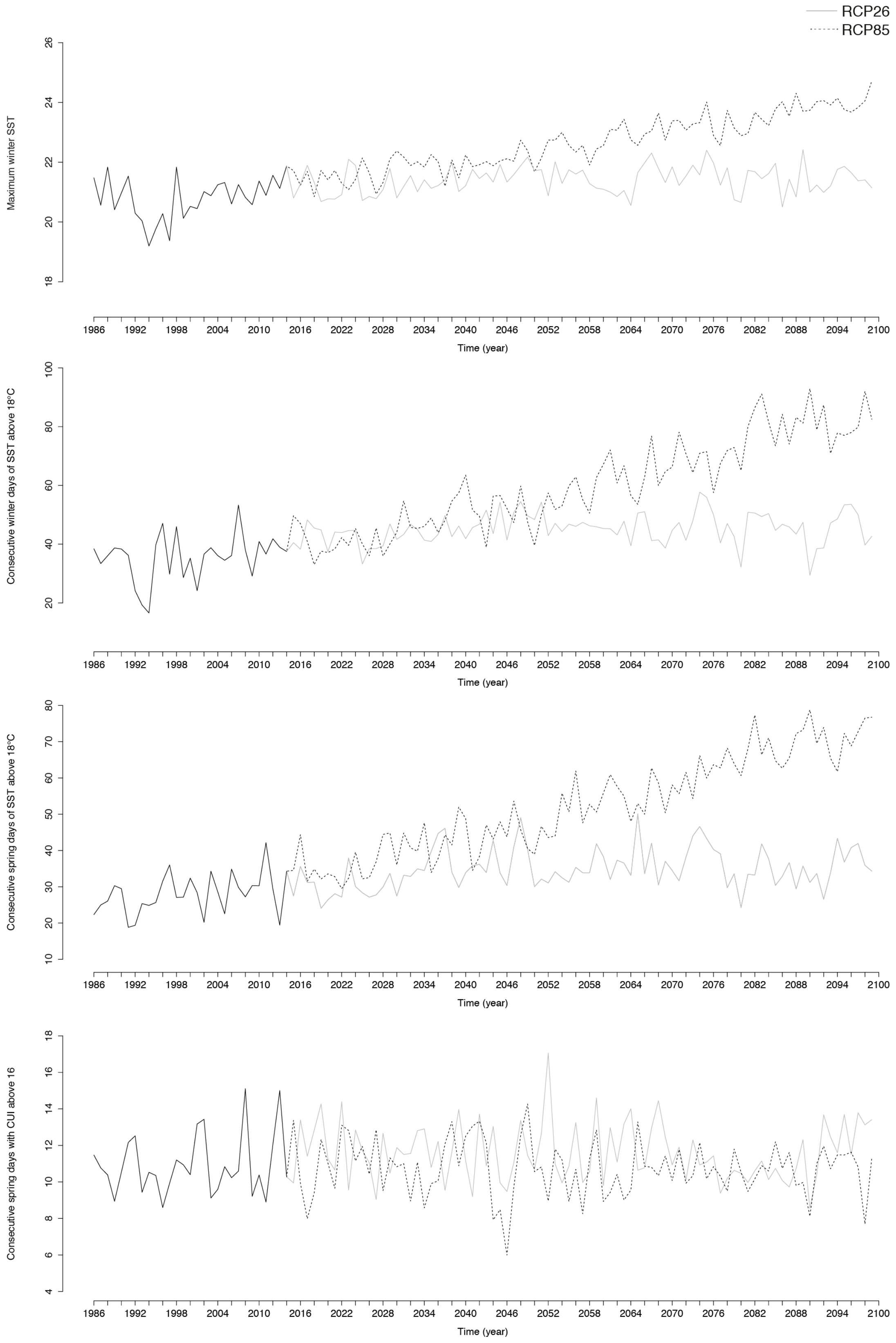

Supplementary Figure S5. Correlation matrix between all environmental predictors used to model the ecological niche of *Saccorhiza polyschides* (year 2010 as an illustrative example). The diagonal shows the histogram and name of each predictor. The right side of the figure shows the Spearman correlation coefficient between pairs of predictors, while the left side displays a scatter plot between pairs.

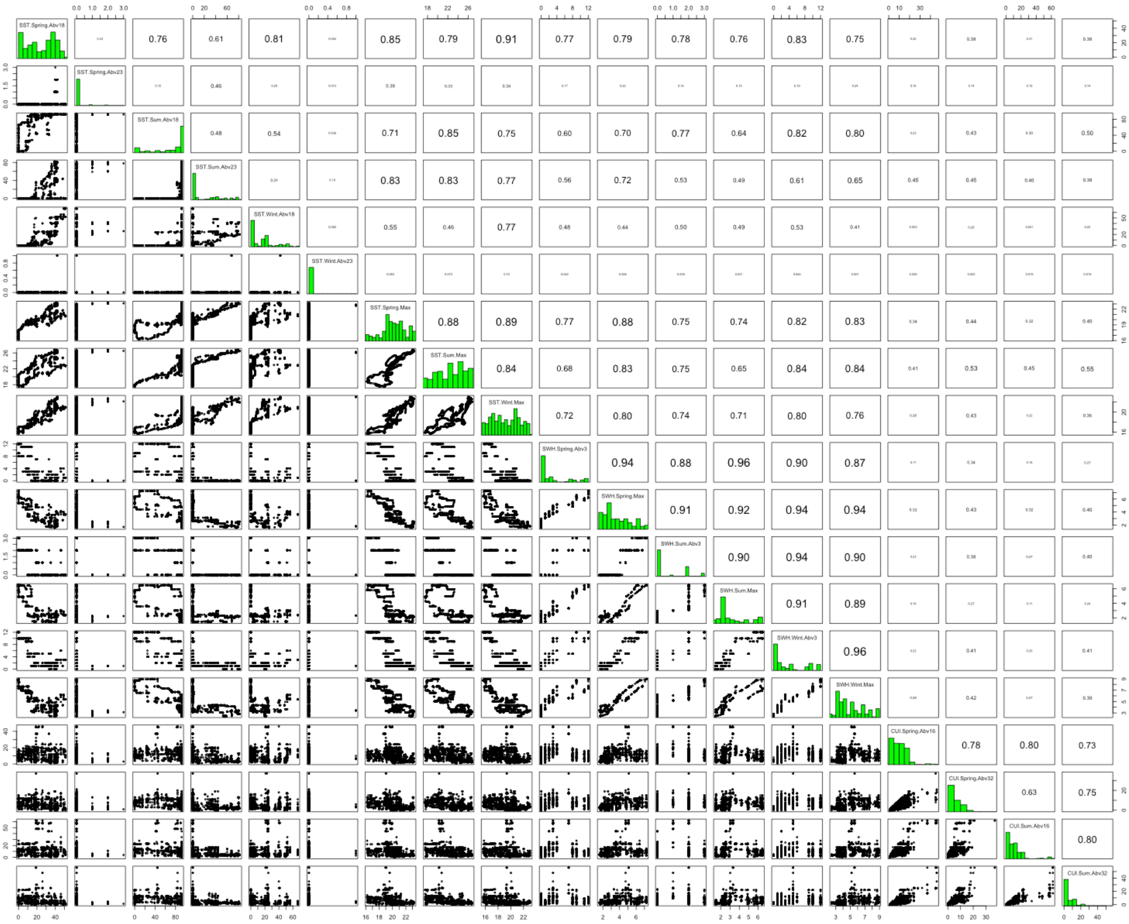

Supplement: Supporting Information [file srep44348-s1.pdf]
